# Deletion of CEP164 in mouse photoreceptors post-ciliogenesis interrupts ciliary intraflagellar transport (IFT)

**DOI:** 10.1371/journal.pgen.1010154

**Published:** 2022-09-08

**Authors:** Michelle Reed, Ken-Ichi Takemaru, Guoxin Ying, Jeanne M. Frederick, Wolfgang Baehr

**Affiliations:** 1 Department of Ophthalmology, University of Utah Health Science Center, Salt Lake City, Utah, United States of America; 2 Stony Brook University, Department of Pharmacological Sciences, Stony Brook, New York, United States of America; 3 Department of Neurobiology & Anatomy, University of Utah, Salt Lake City, Utah, United States of America; 4 Department of Biology, University of Utah, Salt Lake City, Utah, United States of America; Washington University School of Medicine, UNITED STATES

## Abstract

Centrosomal protein of 164 kDa (CEP164) is located at distal appendages of primary cilia and is necessary for basal body (BB) docking to the apical membrane. To investigate the function of photoreceptor CEP164 before and after BB docking, we deleted CEP164 during retina embryonic development (Six3Cre), in postnatal rod photoreceptors (iCre75) and in mature retina using tamoxifen induction (Prom1-ETCre). BBs dock to the cell cortex during postnatal day 6 (P6) to extend a connecting cilium (CC) and an axoneme. P6 retina-specific knockouts (^ret^*Cep164*^-/-^) are unable to dock BBs, thereby preventing formation of CC or outer segments (OSs). In rod-specific knockouts (^rod^*Cep164*^-/-^), Cre expression starts after P7 and CC/OS form. P16 ^rod^*Cep164*^-/-^ rods have nearly normal OS lengths, and maintain OS attachment through P21 despite loss of CEP164. Intraflagellar transport components (IFT88, IFT57 and IFT140) were reduced at P16 ^rod^*Cep164*^-/-^ BBs and CC tips and nearly absent at P21, indicating impaired intraflagellar transport. Nascent OS discs, labeled with a fluorescent dye on P14 and P18 and harvested on P19, showed continued ^rod^*Cep164*^-/-^ disc morphogenesis but absence of P14 discs mid-distally, indicating OS instability. Tamoxifen induction with PROM1^ETCre^;*Cep164*^F/F^ (^tam^Cep164^-/-^) adult mice affected maintenance of both rod and cone OSs. The results suggest that CEP164 is key towards recruitment and stabilization of IFT-B particles at the BB/CC. IFT impairment may be the main driver of ciliary malfunction observed with hypomorphic CEP164 mutations.

## Author summary

CEP164 is indispensable for docking of basal bodies to apical plasma membranes and formation of primary cilia. Homozygous truncations of CEP164 are associated with severe ciliopathy whereas missense mutations generate milder forms of nephronophthisis (NPHP). Recessive mutations of CEP164 are associated with NPHP, Meckel-Gruber (MKS) and Bardet-Biedl syndromes (BBS) in which primary cilia fail to form, or do not function correctly. We found that deletion of CEP164 in mouse photoreceptors before docking of the basal body (BB) to the apical inner segment membrane prevents elaboration of connecting cilia (CC, equivalent to transition zones) and outer segments (OSs, modified primary cilia). We also generated mouse models in which deletion of CEP164 occurs after OSs are established and observed that the BB/CC and axoneme are initially stable in the absence of CEP164. However, prolonged loss of CEP164 resulted in diminished recruitment of intraflagellar transport (IFT) proteins to the BB/CC, thereby affecting IFT which is required for maintenance of cilia and OSs. We propose that, in ciliopathies caused by CEP164 missense mutations (e.g., X1460WextX57 associated with Leber congenital amaurosis or LCA), impairment of IFT provides a mechanism for ciliary dysfunction and disease.

## Introduction

CEP164 (NPHP15) is a ubiquitously expressed centrosomal protein [1] participating in microtubule organization, cell cycle progression and basal body (BB) docking. CEP164 localizes to the BB distal appendages (DAPs) which protrude from the BB distal end as nine-bladed pinwheel-like structures that enable BB docking and ciliogenesis (**S1A and S1B Fig**) [2,3]. Additional key DAP proteins are sodium channel and clathrin linker 1 (SCLT1), centrosomal proteins 83 and 89 (CEP83 and CEP89), Fas-binding factor 1 (FBF1) and C2 domain containing 3 centriole elongation regulator (C2CD3) at the distal BB center, proteins containing multiple coiled-coil domains [2,4]. Super-resolution microscopy demonstrated that CEP164 localizes to the outermost of several concentric rings surrounding the BB distal end [5, 6]. CEP164 recruits tau tubulin kinase 2 (TTBK2) to the BB, an interaction based on the CEP164 WW domain with a TTBK2 proline-rich domain [7]. CEP164-TTBK2 interaction facilitates TTBK2-mediated phosphorylation of both CEP83 [8,9] and M-Phase Phosphoprotein 9 (MPP9) at the distal end of the mother centriole [10]. MPP9 and the associated centrosomal protein CP110-CEP97 complex that caps the distal end of mother centrioles are removed subsequently to promote ciliary axoneme growth [10–12] (**S1C Fig**).

Recessive mutations of CEP164 (e.g., Q11P, R93W, Q525X, R576X, R579X) (**S2B Fig**) are associated with nephronophthisis (NPHP), Meckel-Gruber syndrome (MKS) and renal-retinal ciliopathies [13]. Homozygous truncations of CEP164 are associated with severe ciliopathy whereas missense mutations generate milder forms of NPHP [1,13]. Homozygous CEP164(Q11P) and compound R93W with Q525X are associated with NPHP, while homozygous CEP164(R93W) has been identified in a patient with Bardet-Biedl Syndrome (BBS) [13,14]. Both mutations are outside the WW domain located between residues 56 and 89 [12], but structural analysis revealed that Q11P and R93W mutations compromise TTBK2 complex formation [7]. CEP164(*X1460WextX57*), identified in a child with Leber congenital amaurosis (LCA) [13], causes a read-through of the stop-codon X1460, adding 57 foreign amino acid residues to the CEP164 C-terminus. Biallelic pathogenic variants in CEP164 are causative of oral-facial-digital syndromes (OFDS) [15].

Knockdown of CEP164 in zebrafish resulted in syndromic ciliopathy with ventral body axis curvature, cell death, abnormal heart looping, pronephric tubule cysts, hydrocephalus, and retinal dysplasia [13,16]. Homozygous *Cep164* mouse knockouts were embryonically lethal [17]. In a conditional knockout mouse model that lacks CEP164 in multiciliated tissues and testes, a profound loss of airway, ependymal, and oviduct multicilia resulted, and the mutant mouse developed hydrocephalus and male infertility [17]. Using tracheal cell cultures of this mouse model, CEP164 was shown to regulate small vesicle recruitment, ciliary vesicle formation and BB docking [17].

Photoreceptor outer segments (OSs) are considered modified primary cilia, dedicated to light reception and phototransduction [18–21]. As CEP164 forms a complex with multiple ciliary proteins [22], and there is variance in patient phenotype with CEP164 disruption, additional functions may be revealed depending on depletion before or after ciliogenesis. We generated CEP164 conditional knockouts in retina with three different *Cre* drivers that produce CEP164 truncation during retina embryonic development (^ret^*Cep164*^-/-^) pre-ciliogenesis, in rod photoreceptors (^rod^*Cep164*^-/-^) post-ciliogenesis, and in adult retina using tamoxifen induction (^tam^*Cep164*^-/-^) (**S2A Fig**). ^ret^*Cep164*^-/-^ mice were unable to elaborate photoreceptor connecting cilia (CC) and OSs, whereas ^rod^*Cep164*^-/-^ mice do form photoreceptor CC and OS as *Cre* expression starts post-ciliogenesis. Depletion of CEP164 post-ciliogenesis results in profound loss of intraflagellar transport (IFT) particles, leading to impairment of IFT and OS degeneration. We propose that, in ciliopathies caused by missense mutations (e.g., X1460WextX57 associated with Leber congenital amaurosis (LCA), impairment of IFT provides a mechanism for ciliary dysfunction and disease.

## Results

### Generation of retina-specific *Cep164* knockout mice

The murine *Cep164* gene (30 exons, one noncoding exon 1) produces multiple splice variants lacking internal exons. The main variant encodes a protein of 1333 amino acids (150 kDa) carrying an N-terminal WW domain and three coiled-coil domains. To generate a retina-specific knockout mouse at various stages of development (**S2A Fig**), *Cep164*^F/F^ mice [17] were bred with transgenic Six3Cre mice (^ret^*Cep164*^-/-^) [23], iCre75 mice (^rod^*Cep164*^-/-^) [24], or Prom1(C-L) (Prom1-ETCre) (^tam^*Cep164*^-/-^) mice [25] to produce null alleles (**S2C and S2D Fig**). Six3 (*sine oculis*-related homeobox 3) is a transcription factor expressed in retina, RPE and brain during embryonic development [23]. *Cre* in iCre75 is under control of the rhodopsin promoter that expresses in the second postnatal week, allowing CC and OS to develop. Tamoxifen induction allows depletion of CEP164 at any time during development, including in the adult mouse. LoxP sites placed in introns 3 and 4 specify deletion of exon 4 (**S2C Fig**), truncating CEP164 after exon 3 (after residue Pro 64 inside the WW domain) as exon 5 is out-of-frame. Truncation after exon 3 is predicted to eliminate all splice variants and the WW domain interacting with TTBK2. Genotyping with tail and retina DNA as templates and primers flanking exon 4 verified knockout of the *Cep164* gene (**S3 Fig**). Immunohistochemistry in P21 wildtype (WT) retina with anti-CEP164 antibody showed that CEP164 is a photoreceptor inner segment (IS) protein (**S1A Fig**) and accumulates at the photoreceptor BB, identified by EGFP-CETN2 (**S1B Fig).**

### ^ret^*Cep164*^-/-^ photoreceptors lack connecting cilia and outer segments

**Fig 1:**
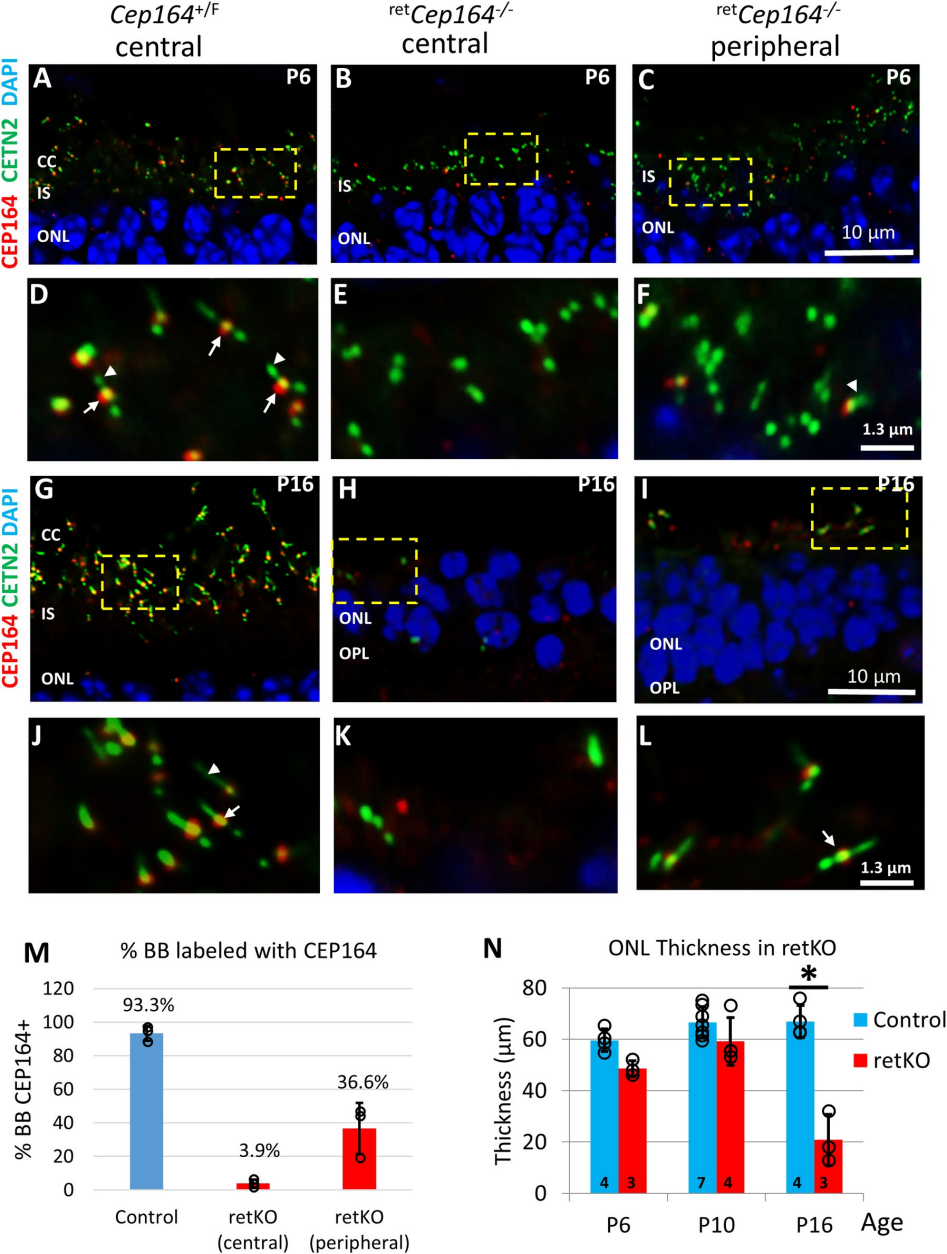
Rapid postnatal ^ret^*Cep164*^-/-^ photoreceptor degeneration. **A-C**, P6 *Cep164*^+/F^ (A), ^ret^*Cep164*^*-/-*^ central (B) and peripheral (C) cryosections on Egfp-Cetn2^+^ background probed with anti-CEP164 antibody (red). In ^ret^*Cep164*^*-*/-^ cryosections, CEP164-BB association is much reduced, but not in retina periphery where degeneration is delayed. **D-F**, enlargements of A-C as indicated. CEP164 associates with the BB (arrows) in control sections (D), is absent in the knockout (B’), but detectable at some BB in the retina periphery (F). Arrowheads identify CC. **G-I**, P16 cryosections probed with anti-CEP164 antibody. Note reduced thickness of mutant ONL. **J-L**, enlargements as indicated in G-I. At P16, control CC (G, J) are normal length and CEP164 is firmly associated with the BB (arrows in J). In central ^ret^*Cep164*^*-*/-^ sections (E), ONL is reduced to 1–3 nuclear rows, BBs and CCs have disintegrated. In the peripheral retina (F), few BB structures survive (arrow in L). **M**, % BBs in P6 control, central and peripheral ^ret^*Cep164*^*-/-*^photoreceptors. In P6 ^ret^*Cep164*^*-*/-^ retina cryosections, CEP164 association with BB is much reduced compared to CEP164^+/F^ control (3.9–36.6%) and CC are not formed. Over 100 BB were counted per each animal across three controls and 3 ^ret^*Cep164*^*-/-*^. **N**, ONL thickness at P6, P10 and P16 in central ^ret^*Cep164*^*-/-*^ (n = 3–7). Note significant decrease at P16. * p<0.05, *t*-test.

Six3*Cre* expression begins at embryonic day 9 (E9) and the floxed gene of interest is deleted subsequently. In P6 *Cep164*^*F/+*^;Egfp-Cetn2 controls, BBs docked to the cell cortex as evidenced by presence of CC distal to BBs (**Fig 1A**). CEP164 appears firmly attached to BBs (**Fig 1D**, arrows) and short CC are formed (**Fig 1D**, arrowheads). Few ^ret^*Cep164*^-/-^ BBs carry CEP164 and CC formation declines in central ^ret^*Cep164*^-/-^ photoreceptors (**Fig 1B and 1E**), presumably because BBs are unable to dock to the IS apical membrane. Similarly, in primary cultures of tracheal multiciliated cells from *Cep164*^F/F^;FoxJ1-Cre mice, up to 83% of BBs were undocked [17]. As Six3Cre expression generates a central-to-peripheral gradient of gene product depletion [26], knockout is delayed in peripheral ^ret^*Cep164*^*-/-*^ retina (**Fig 1C**), and consequently some photoreceptors develop short CC (about 1/3) (**Fig 1F**, arrowhead). Mother centrioles with bound CEP164 (**Fig 1J**, arrow) extend fully developed CC in P16 control photoreceptors (**Fig 1G and 1J**, arrowhead), whereas in central knockout retina, BBs disintegrate and photoreceptors degenerate (**Fig 1H** and **1K**). Few BBs with CC survive in the retina periphery (**Fig 1I** and **1L,** arrow in L). Quantitative evaluation reveals that 93.3% control BBs, but only 3.9% knockout BBs, were labeled by antibody directed against CEP164 in P6 central retina (n = 3, **Fig 1M**). Labeling increased in the peripheral retina to 36.6%. Absence of 100% labeling in the control is attributed to the imaging plane since CC formation was robust. Thickness of ^ret^*Cep164*^-/-^ outer nuclear layer (ONL) appears to be stable at P6 and P10, but declines by P16 (n = 3, **Fig 1N**).

### ^ret^*Cep164*^*-/-*^ photoreceptor function at P16 and P25

**Fig 2:**
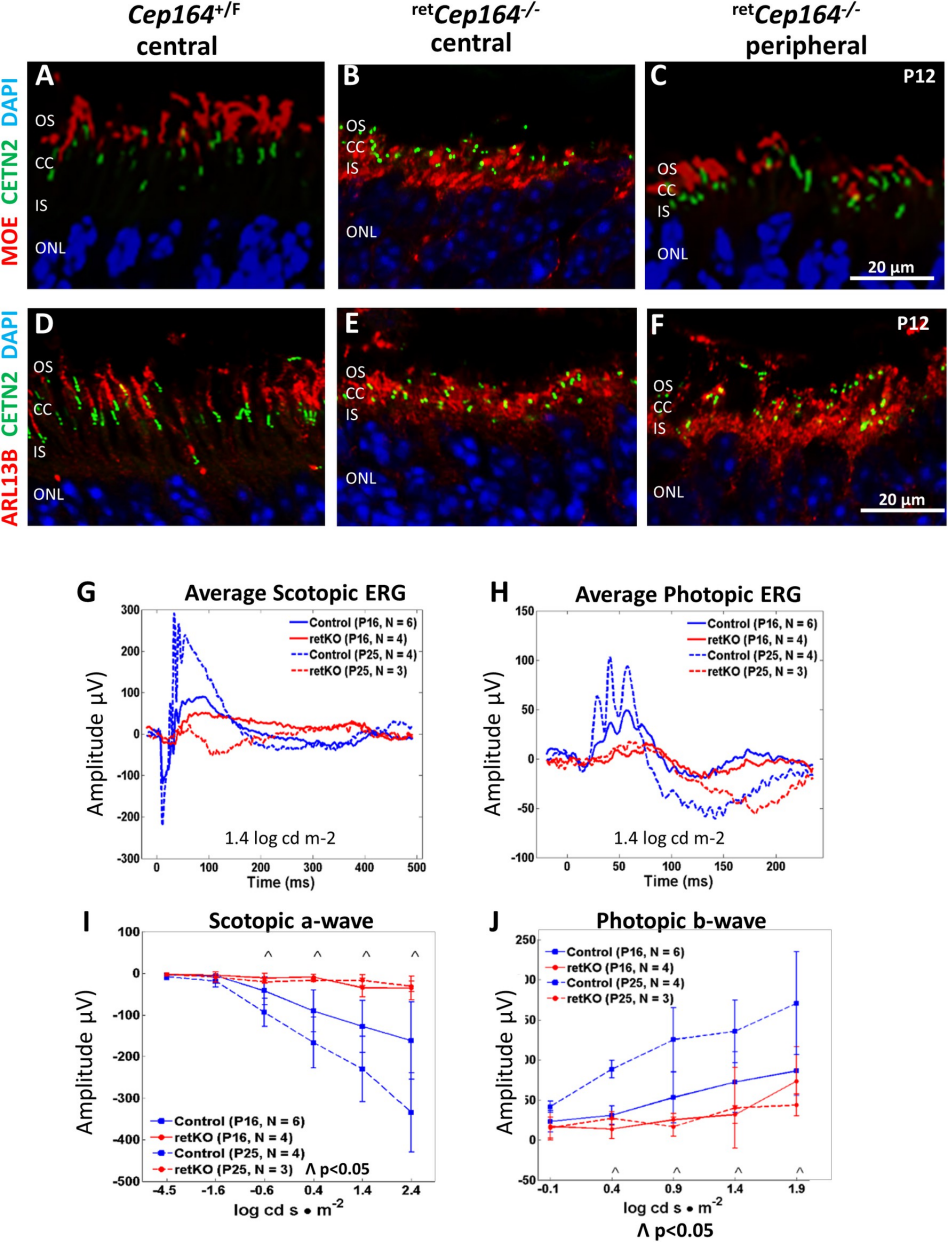
Central ^ret^*Cep164*^-/-^ photoreceptors do not form OSs. **A-C**, P12 central *Cep164*^+/F^ (A), central ^ret^*Cep164*^*-/-*^ (B) and peripheral ^ret^*Cep164*^*-/-*^ retina cryosections(C) probed with MOE (anti-PDE6) (red). PDE6 is an OS protein participating in phototransduction. In central ^ret^*Cep164*^*-/-*^ sections (B), OSs are not formed and PDE6 mislocalizes to the IS. In retina periphery (C), degeneration is delayed and some OSs are present. **D-F**, cryosections as in A-C probed with anti-ARL13B, a small GTPase present in the OS. In controls (D), ARL13B correctly localizes to OSs, whereas it mislocalizes to photoreceptor IS of central and peripheral ^ret^*Cep164*^*-/-*^ retina. **G-J**, Electroretinography (ERG) at P16, P25. Average scotopic ERG (G) and photopic ERG (H) in control (blue) and retina knockouts (red) at 1.4 log cd s m^-2^. Scotopic a- wave (I) and photopic b-wave (J) amplitudes (μV) as a function of light intensity (log cd s m^-2^). Scotopic ERGs show diminished responses by P16 and statistically different responses (^, p < .05) at P25. Photopic ERGs show diminished b-wave responses by P16 and statistically different responses (^, p < .05) at P25. Minimal scotopic a- and b-waves reveal surviving photoreceptors of retina periphery.

Antibodies directed against PDE6 and ARL13B were used to assay for OS elaboration in central versus peripheral retina. PDE6 is a key phototransduction enzyme [27] and ARL13B is a small GTPase functioning as a guanine nucleotide exchange factor (GEF) of ARL3 [28]. In cryosections of *Cep164*^+/F^;Egfp-Cetn2 control retina, PDE6 (MOE antibody) and ARL13B localize in the photoreceptor OS as expected (**Fig 2A** and **2D**). In central ^ret^*Cep164*^*-/-*^ sections, PDE6 (**Fig 2B**) and ARL13B (**Fig 2E**) mislocalize to the IS as OSs fail to form. In peripheral retina (**Fig 2C**), PDE6 localizes to rudimentary OSs while ARL13B remains mostly confined to IS (**Fig 2F**). OS formation in the retina periphery encouraged functional analysis by pan-retina electroetinography (ERG) allowing assessment of photoreceptor function in dark- and light-adapted retina [29]. In response to light, photoreceptors hyperpolarize, causing a change in electric activity that can be detected by ERG, a diagnostic test called employing light flashes of various intensity [29]. Scotopic a-wave (rod function) and photopic b-wave amplitudes (cone and bipolar cell function) were assessed with flashes of 1.4 log cd s·m^-2^. Scotopic and photopic responses were almost extinguished at P16 and P25 indicating loss of rod and cone function (n = 3–6, **Fig 2G** and **2H**). High flash intensities under scotopic conditions elicit weak a-wave responses (**Fig 2I**) and under photopic conditions an even stronger response (**Fig 2J**) consistent with minor OS formation and photoreceptor survival at the retina periphery.

### *Cep164* rod knockout

Previous reports dealt with CEP164’s role in early steps of ciliogenesis, such as preciliary vesicle recruitment and BB docking, but its role in ciliary maintenance is unknown. To determine whether CEP164 is required once CC are established, we examined deficits in a rod-specific knockout of CEP164 driven by iCre75 under control of the rhodopsin promoter (**S2A Fig**). One advantage of a rod-specific knockout is that *Cre* expression does not occur before P7, thus permitting ciliogenesis and OS formation. We asked, whether CEP164 is necessary for continued rod viability and, if so, how fast rods would degenerate after CEP164 deletion. Genotyping of ^rod^*Cep164*^*+/-*^ control and *Cep164*^F/F^;iCre75 (abbreviated ^rod^*Cep164*^*-/-*^) retina DNA confirmed that *Cep164* exon 3 excision was complete at P16 (**S3G Fig**, lane 5).

### ^rod^*Cep164*^*-/-*^ outer segment instability

**Fig 3:**
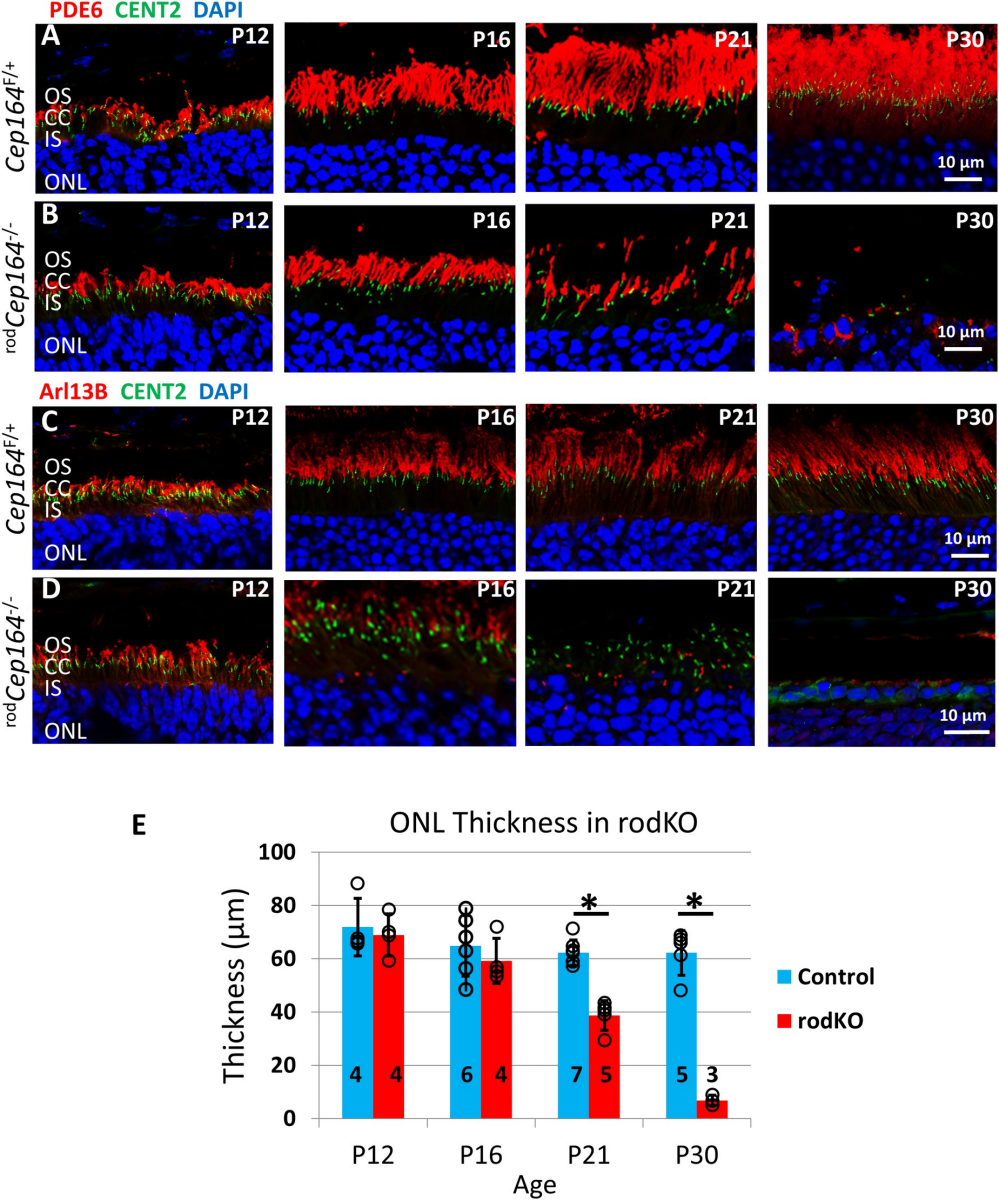
^rod^*Cep164*^-/-^ OSs degenerate after P16. Rows **A, B,** *Cep164*^F/+^ (A) and *Cep164*^*F/F*^;iCre75 (^rod^*Cep164*^-/-^) retina cryosections (B) probed with PDE6 antibody (MOE, red) (A, B) at P12, P16, P21 and P30. All sections are on Egfp-Cetn2 background to mark the position of centrioles and CC. Note disintegrating ^rod^*Cep164*^-/-^ OSs in B at P21 where CC (green) is still congruent with the OS (red). Rows **C, D**, central *Cep164*^+/F^ (C) and central ^rod^*Cep164*^*-/-*^ (D) retina cryosections probed with anti-ARL13B (red) at P16, P21 and P30. Note ARL13B, known to interact with CEP164, does not reach the OS in the absence of CEP164. **E**, ONL thickness at P12, P16, P21 and P30 in central retina of ^rod^*Cep164*^-/-^ (n = 3–7). ONL thickness is indistinguishable at P12 and P16, but decreases rapidly after P16. * p<0.05, *t*-test.

Depletion of CEP164 after P16 raises the question of BB-membrane attachment and OS stability. Plastic sections of P16 ^rod^*Cep164*^*-/-*^ photoreceptors demonstrated that IS/OS length and ONL thickness are comparable to heterozygous control (**S4A Fig**), but are reduced at P21 (**S4B Fig**). We again used anti-PDE6 (**Fig 3**, rows **A** and **B**) and anti-ARL13B antibodies (**Fig 3**, rows **C** and **D**) to determine OS stability by immunohistochemistry. At P12, control and ^rod^*Cep164*^*-/-*^ are comparable and called “predegenerate.” At P16, ^rod^*Cep164*^*-/-*^ OSs appear still stable and ^rod^*Cep164*^*-/-*^ ONL thickness was not statistically different (n = 4–6, **Fig 3E**). However, by P21, OSs became unstable and the ONL thickness was reduced by one-third (n = 5–7, **Fig 3B** and **3E**). By P30, ^rod^*Cep164*^*-/-*^ OSs are undetectable and ONL is reduced to a single row of nuclei. At P16, ARL13B is barely detectable in the ^rod^*Cep164*^*-/-*^ OSs and is absent in the mutant OSs at P21 (**Fig 3D**), suggesting that CEP164 is necessary for ARL13B to reach the OS. While the ^rod^*Cep164*^*-/-*^ ONL was not significantly reduced at P12 and P16 relative to control ONLs (n = 3–7, **Fig 3E**), the P16 ^ret^*Cep164*^*-/-*^ ONL thickness was less than half compared to control consistent with distinct earlier onset of Cre-based recombination (n = 3–4, **Fig 1N**).

### Loss of CEP164 at ^rod^*Cep164*^*-/-*^ BB

**Fig 4:**
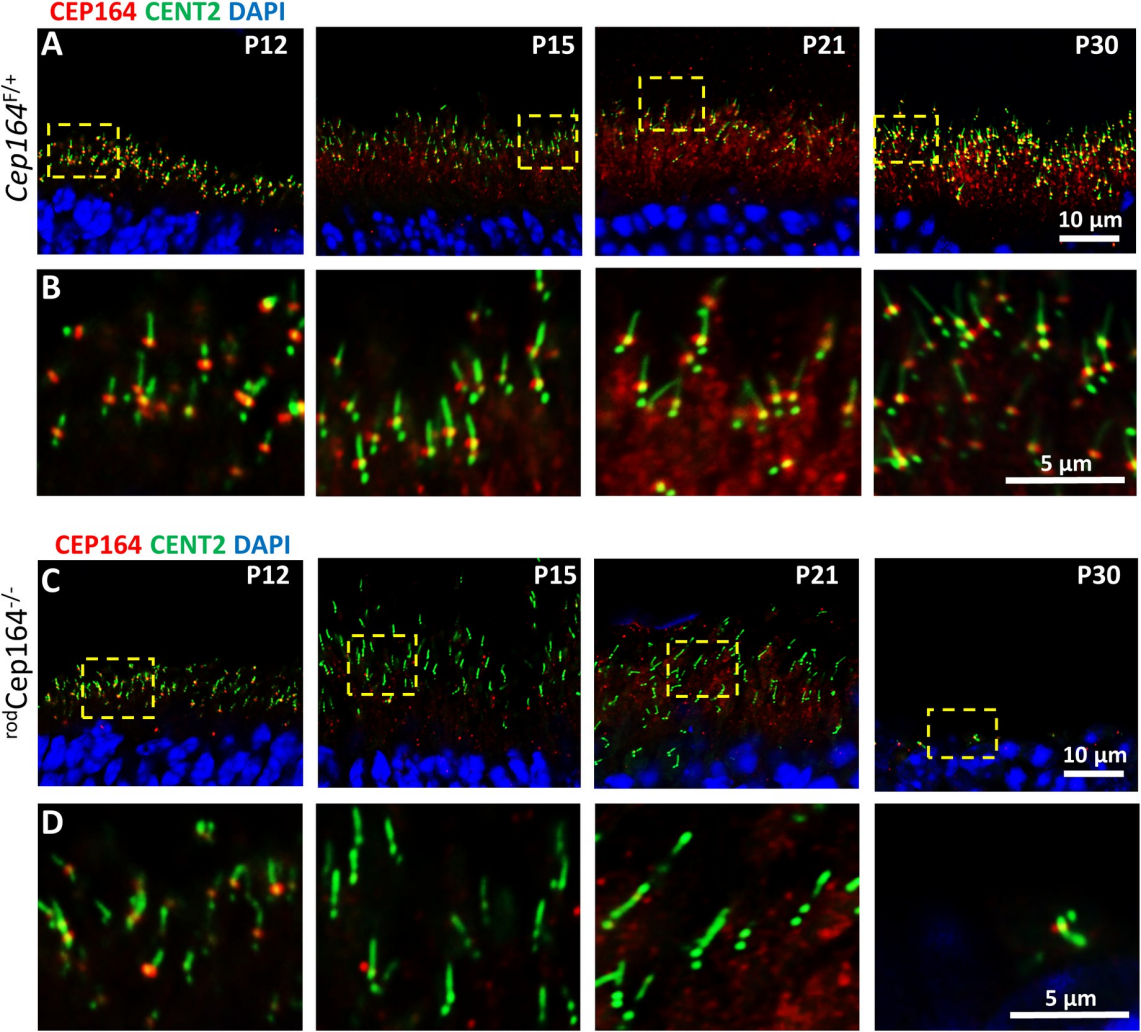
Cep164 rod knockout BB/CC stability. Row **A,** *Cep164*^+/F^; iCre75 (^rod^*Cep164*^+/F^) retina cryosections on Egfp-Cetn2^+^ background probed with CEP164 antibody (red) at P12, P16, P21 and P30. Row **B**, enlargements as indicated by dashed squares in row A. Row **C**, ^rod^*Cep164*^-/-^ cryosections probed with CEP164 antibody. Row **D**, enlargements as indicated by dashed squares in row C. Note CEP164 is attached to ^rod^*Cep164*^-/-^ BB at P12, but lost at P15 and P21. BB and CC appear perfectly normal at P15 and P21 in the absence of CEP164. At P30, rods are degenerated. Scale bar, 10 μm in rows A and C, 5 μm in rows B and D.

**Fig 5:**
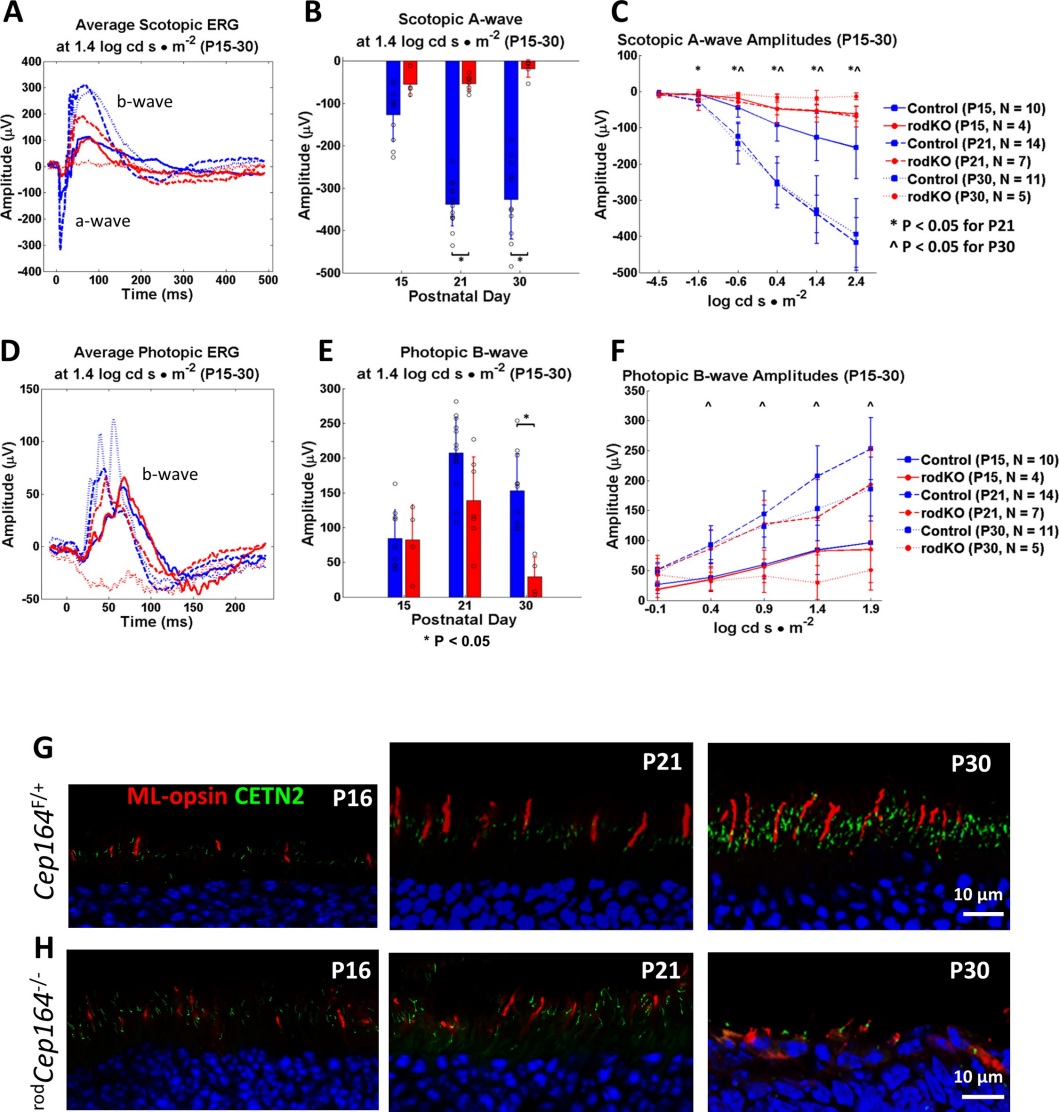
^rod^*Cep164*^-/-^ scotopic and photopic ERG and fate of cones. **A,** control (blue) and ^rod^*Cep164*^-/-^ mice (red) were dark-adapted overnight and exposed under scotopic conditions to flashes of 1.4 log cd s/m^2^ at P16, P21 and P30 (n = 4–14). Note knockout a- and b- wave amplitudes were diminished at all ages**. B,** statistical evaluation of amplitudes of control and knockout CEP164 scotopic ERG a-waves at P15, P21 and P30 as measured in A. At P21 and P30 * p<0.05 *t*-test **C, F,** scotopic a-wave (C) and photopic b-wave amplitudes (F) at P16, P21 and P30 as a function of light intensity. Knockout scotopic a- and b-waves are diminished, but never extinguished. * p<0.05 at P21 and ^ p<0.05 at P30. **D,** control (blue) and ^rod^*Cep164*^-/-^ mice (red) were exposed under photopic conditions to flashes of 1.4 log cd s/m^2^. Note, ^rod^*Cep164*^-/-^ photopic b-waves were absent at P30. **E,** statistical evaluation of photopic b-wave amplitudes at 1.4 log cd s/m^2^ as measured in B. **G, H,** immunohistochemistry with anti-ML-opsin of control (G) and ^rod^*Cep164*^-/-^ central cryosections (H) at P16, P21 and P30. Cones on the ^rod^*Cep164*^-/-^ background are stable until P21 but are degenerated at P30.

Immunohistochemistry at P12, P15, P21 and P30 revealed that CEP164 was firmly attached to the BB distal appendages in *Cep164*^+/F^;Egfp-Cetn2 cryosections (**Fig 4**, rows **A** and **B**). At P12, ^rod^*Cep164*^*-/-*^ BBs reveal normal appearance with CEP164 firmly attached. At P15 most BBs have lost most CEP164 and at P21, CEP164 was undetectable at BBs (**Fig 4**, rows **C** and **D**). The P30 knockout ONL revealed one layer of nuclei; a single BB/CC structure is likely that of a surviving cone (**Fig 4D,** P30), see also **Fig 5H,** P30).

### ^rod^*Cep164*^*-/-*^ photoreceptor function

Scotopic (rod function) and photopic (cone function) pan-retina ERGs were performed at a constant 1.4 log cd s/m^2^ intensity and the traces averaged (N = 4–14) (**Fig 5A** and **5D**). In scotopic ERG with control animals, rod a-waves continue to increase from P15 (120 μV) to P30 (300 μV) while ^rod^*Cep164*^-/-^ rod a-waves hover at 50 μV at P16 and P21 (**Fig 5A** and **5B**). At P30, the ^rod^*Cep164*^-/-^ rod a-wave is nearly extinguished, consistent with absence of rods (**Fig 5B**). The photopic ^rod^*Cep164*^*-/-*^ cone b-wave is identical to control at P15, reduced at P21 and extinguished at P30, indicating cone degeneration (**Fig 5D** and **5E**). This is consistent with previous findings where deletion of rod-specific genes resulted in secondary loss of cones due to diminished rod-derived cone viability factor (RdCVF) [30]. Scotopic ERG as a function of light intensity showed diminished rod a- and b-wave amplitudes as early as P15 beginning at 1.4 log cd s/m^2^ (**Fig 5B** and **5C**) suggesting rod degeneration has begun, although not statistically significant at that age. Photopic cone b-wave amplitudes of control and rod knockout mice were identical at P15 but those of knockouts were nearly extinguished by P30 (**Fig 5E** and **5F**). Assuming that cone bipolar cells are unaffected in ^rod^*Cep164*^-/-^ retinas, the severely attenuated photopic cone b-wave derives from loss of cone function. Cone degeneration in the rod knockout was verified using anti-ML-opsin antibody (**Fig 5G** and **5H**). P21 ^rod^*Cep164*^-/-^ cones are relatively stable, but disintegrate by P30.

### Loss of IFT88, IFT57 and IFT140 at BB and CC

**Fig 6:**
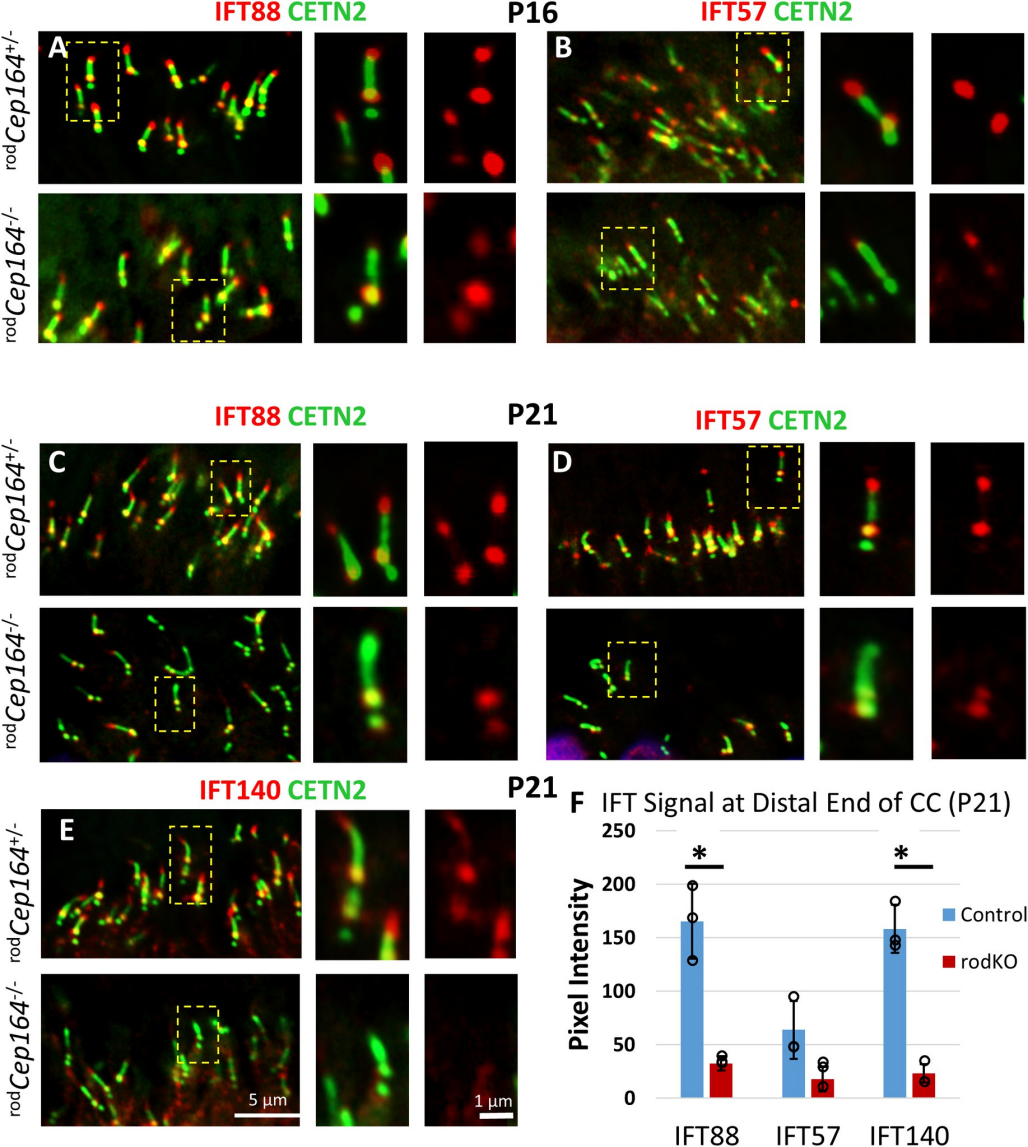
Loss of IFT88, IFT57 and IFT140 in ^rod^*Cep164*^-/-^ photoreceptors. **A, B,** immunohistochemistry of ^rod^*Cep164*^+/-^ (upper panels) and ^rod^*Cep164*^-/-^ (lower panels) cryosections at P16 probed with anti-IFT88 (A) and anti-IFT57 (B) antibodies. **C-E**, immunohistochemistry of P21 ^rod^*Cep164*^+/-^ (upper panels) and ^rod^*Cep164*^-/-^ (lower panels) cryosections probed with anti-IFT88 (C), anti-IFT57 (D) and anti IFT140 (E). Insets to the right of main panels show enlargements of individual representative BB/CC structures as indicated by hatched rectangles (left panels) or antibody alone (red). **F**, red channel pixel intensity of IFT88, IFT57 and IFT140 signal at CC distal end at P21 using 3 animals each. The IFT57 signal was not significant, likely due to higher variance in the control.

**Fig 7:**
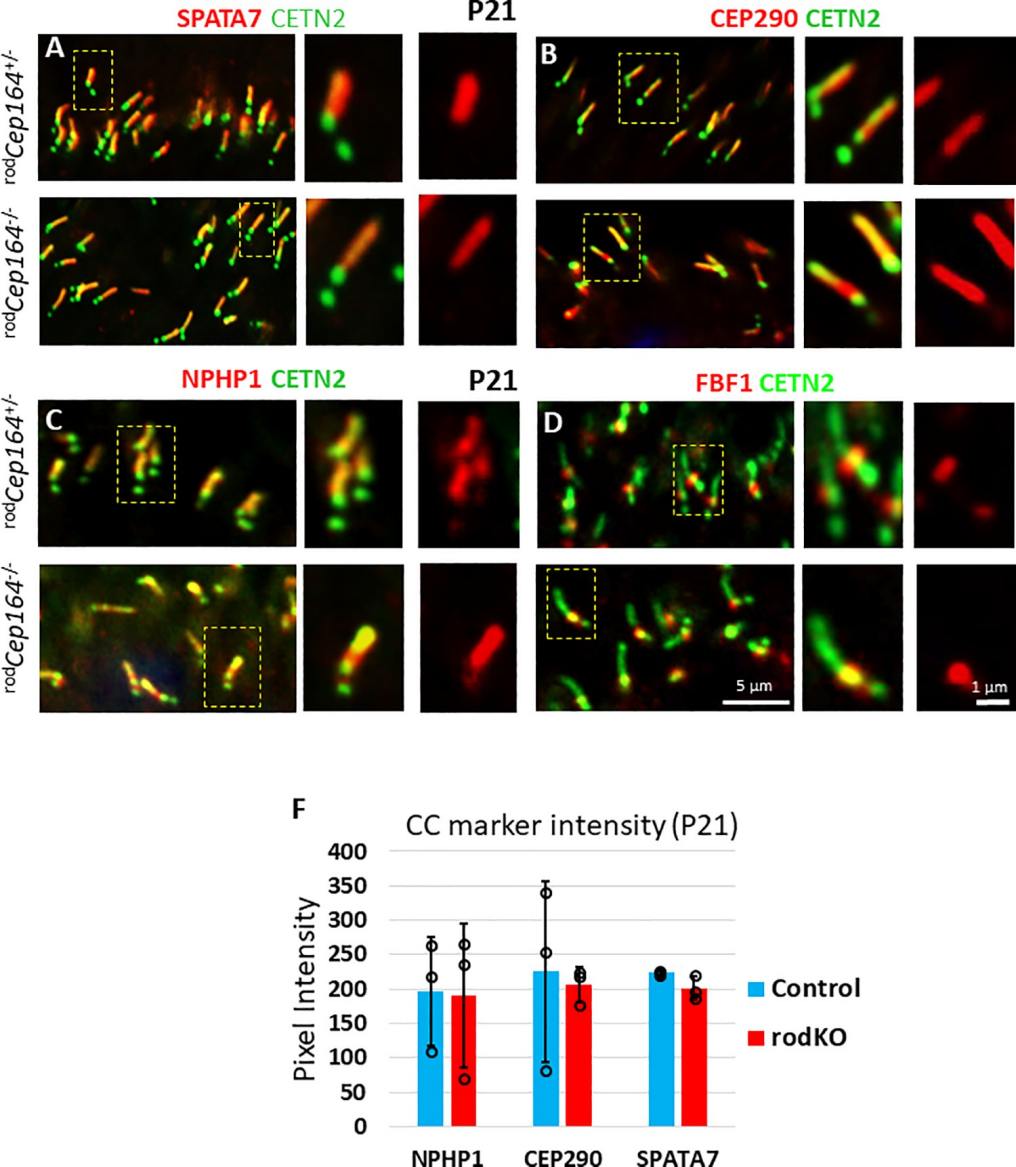
SPATA7, CEP290, NPHP1 and FBF1 at the ^rod^*Cep164*^-/-^ CC or BB. **A-D,** immunohistochemistry of ^rod^*Cep164*^+/-^ (upper panels) and ^rod^*Cep164*^-/-^ (lower panels) cryosections probed with at P21 probed with anti-SPATA7 (A), anti-CEP290 (B), with anti-NPHP1 (E), and anti-FBF1 (D) antibodies. Note no relevant changes for SPATA7, CEP290, NPHP1 and FBF1 localizations. Insets (right) show enlargements of representative BB/CC structures identified by Egfp-CETN2 or individual antibodies (red). **F,** red channel pixel intensity of NPHP1, CEP290 and SPATA7 signal at CC at P21 using 3 animals each. None are statistically significant. p>0.05, *t*-test.

**Fig 8:**
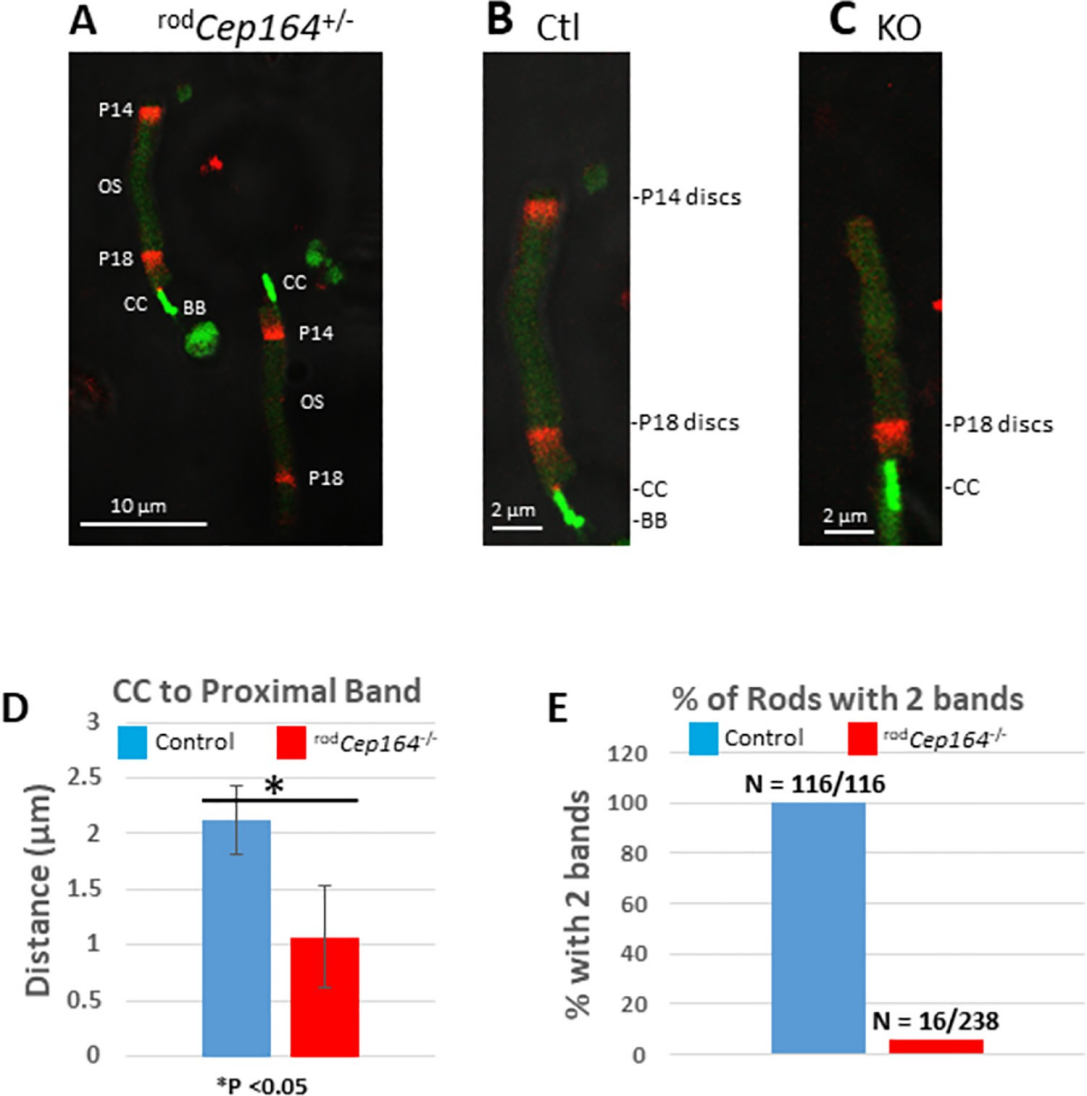
Intravitreal CF-568 hydrazide dye injection. Dye was injected at P14 (day1) and P18 (day 4) into ^rod^*Cep164*^+/-^control versus ^rod^*Cep164*^-/-^ eyes on Egfp-Cetn2^+^ background and retinas were collected at P19 and dissociated rods were imaged. **A**, panel of ^rod^*Cep164*^+/-^ control rods. **B**, single enlarged ^rod^*Cep164*^+/-^ rod with P14 (day 4) and P18 (day 1) disc bands. **C**, single ^rod^*Cep164*^-/-^ rod with new P18 discs, but no P14 discs. **D**, statistical evaluation of the distance between CC-tip and center of P18 band in control (blue) and rod KO (red) (n = >100), * p<0.05 *t*-test. **E**, % rods with P14 and P18 bands in control versus mutant (n = >100).

In primary cilia (RPE1 cells), CEP164 recruits NPHP1 and IFT88 to the mother centriole and, upon deletion of CEP164, the levels of BB-associated IFT88 strongly decrease [31]. In contrast to primary cilia, CEP164 is dispensable for the recruitment of IFT components to multicilia [17]. We explored whether IFT88, IFT57 and IFT140 levels vary in P16 ^rod^*Cep164*^-/-^ retinas (**Fig 6A–6E**) before onset of photoreceptor cell and OS degeneration, and at P21 when OSs disintegrate and ONL thickness is reduced (see **Fig 3**). P16 and P21 control ^rod^*Cep164*^+/-^ photoreceptors reveal the familiar pattern of IFT proteins positioned at the BB and CC distal tips (**Fig 6A–6E),** upper panels) [32–34]. By contrast, P16 ^rod^*Cep164*^-/-^ photoreceptors reveal reduced levels of IFT proteins at BB and CC, although not statistically significant (n = 3, **Fig 6A** and **6B,** lower panels, **S5 Fig**), and IFT protein levels are significantly attenuated in P21 knockouts (n = 3, **Fig 6C** and **6D,** lower panels, **Fig 6F**). IFT88, IFT57 and IFT140 fluorescence signal intensities at distal CC in ^rod^*Cep164*^-/-^ retina are reduced at P16, although not statistically significant (**S5 Fig**), and to 30% or less by P21 (**Fig 6F**). SPATA7 (spermatogenesis associated protein 7) [35] and CEP290 [36,37] are unaffected by deletion of CEP164 (**Fig 7A, 7B** and **7F**) suggesting normal CC elaboration. NPHP1 remains unaffected by post-ciliogenesis deletion of CEP164 (**Fig 7C** and **7F**). FBF1 remains at the BB in the ^rod^*Cep164*^*-/-*^, suggesting intact DAPs (**Fig 7D**). These results suggest that CEP164 recruits IFT88, IFT140 and IFT57 to photoreceptor BB/CC, as observed in primary cilia [31]. IFT88 and IFT57 protein levels are reduced in the ^rod^Cep164^-/-^, negatively affecting IFT and OS axoneme stability as evidenced by shortening of ^rod^*Cep164*^*-/-*^ rod OSs at P16 and P21 (**Fig 3B** and **3D**) and absence of P14 discs in the dye injection experiment (see below, **Fig 8C**).

Distributions of two BB-associated proteins, pericentrin (PCNT) and ninein (NIN), were examined in ^rod^*Cep164*^-/-^ photoreceptors. PCNT interacts with numerous proteins including IFT proteins [38], the γ-tubulin ring complex [39], cytoplasmic dynein [40], PCM1 [41] and CEP215 [42], stabilizing microtubules and/or aiding microtubule nucleation [39]. PCNT localizes to the pericentriolar material (PCM) surrounding the BB of mouse photoreceptors [39,43]. Immunohistochemistry reveals that PCNT trails from the BB presumably along microtubules (**S6A Fig**), upper panel); PCNT localization of P21 ^rod^*Cep164*^*-/-*^ BB is unchanged but slightly reduced (**S6A Fig,** lower panel). In cultured cells, ninein colocalizes with γ-tubulin and C-Nap1, also known as CEP250 [44], and is important for positioning/anchoring the microtubule minus-ends to BB subdistal appendages [45]. Surprisingly, ninein localizes only weakly to the BB of control photoreceptors (**S6B** and **S6C Fig,** upper panels), but labels robustly the centrioles of ^rod^*Cep164*^*-/-*^ photoreceptors (**S6B** and **S6C Fig,** lower panels), using two distinct antibodies (Abclonal or Sigma Aldrich). One explanation of the differential BB labeling is masking of the ninein epitope by another BB protein the localization of which is altered in the *Cep164*^-/-^ rod. The significance of labeling of the proximal OSs and the rootletin/microtubule area (arrows in **S6B** and **S6C Fig**, upper panels) is unclear, but ninein was previously shown to be released from the centrosome to move along microtubules in epithelial cells providing a potential explanation [46].

### Disc formation in ^rod^*Cep164*^*-/-*^ photoreceptors

All ~800 discs of the mouse OS are renewed every ten days and roughly 80 discs are synthesized every day, moving towards the retina pigmented epithelium (RPE) to be phagocytosed [47]. To identify mechanisms that cause OS degeneration more precisely, we developed a fluorescent dye assay to monitor the formation of closed discs during disc morphogenesis and their fate. As Cre expression varies in each photoreceptor, to some extent, and dye sequesters into new discs in OS per photoreceptor, dissociated photoreceptors were examined independently. CF-568-hydrazide, a water-soluble, membrane impermeable, aldehyde-fixable red-fluorescent dye [48], was injected intravitreally on P14 (day 1) and P18 (day 4) and eyes were harvested on P19 (day 5). Following retina dissociation, ^rod^*Cep164*^+/-^ control photoreceptors show two bands containing red fluorescent discs along the OS (**Figs 8A** and **3B**) indicating that CF-568 sequestered into the lumen during enclosure of a nascent disc. The two bands are separated by about 10 μm or ~300 discs. Nascent day 1 discs (P18) are located immediately adjacent to the CC (green, identified by EGFP-CETN2), whereas older day 4 discs (P14) are displaced distally towards the retinal pigment epithelium (RPE), as shown by Richard Young [49,50]. Disc morphogenesis occurs as late as day 4 (P18) in ^rod^*Cep164*^-/-^ OSs, albeit slower than controls as newly formed discs appear closer to the distal CC (n = >100, **Fig 8C** and **8D**). Relative to control OSs, day 4 ^rod^*Cep164*^-/-^ bands are very faint or absent, i.e., only ~5% of KO rods display P14 bands (n = >100, **Fig 8E**).

### CEP164 deletion by tamoxifen induction

**Fig 9:**
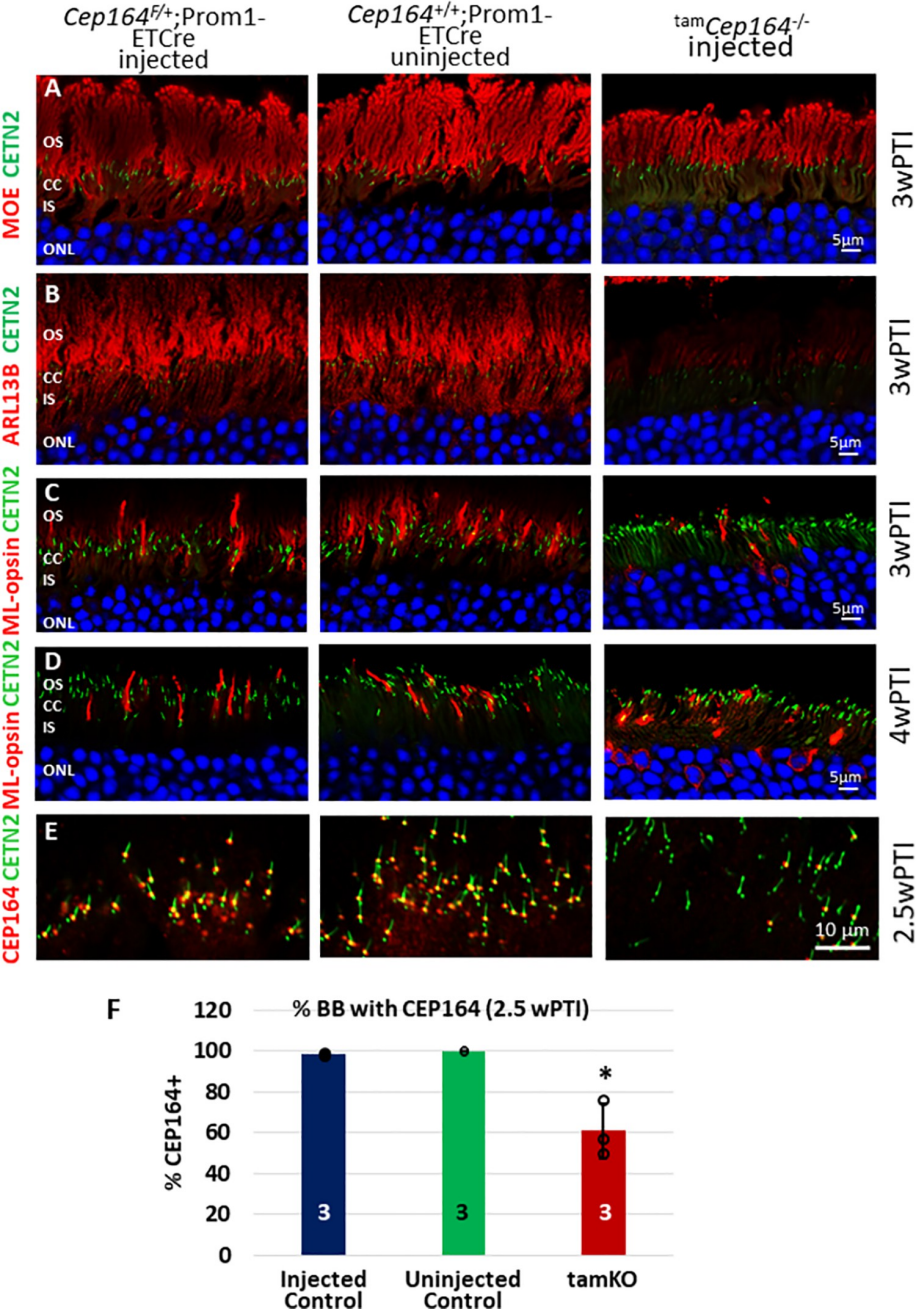
Tamoxifen-induced deletion of CEP164 in adult mouse. Row **A**, *Cep164*^+/F^;Prom1-ETCre (injected) (left panel), *Cep164*^+/+^;Prom1-ETCre (uninjected) (middle panel) and *Cep164*^*F*/F^;Prom1-ETCre (^tam^*Cep164*^-/-^) (injected) (right panels) on EGFP-CETN2 background probed with anti-CEP164. Note near even distribution BB/CCs with and without CEP164 in the knockout panel. Rows **B-E**, immunohistochemistry of injected control cryosections (left panels), of uninjected Prom1-ETCre cryosections (middle panels) and of ^tam^*Cep164*^-/-^ (right panels) probed with MOE (anti-PDE6) (A), anti-ARL13B (B), or ML-opsin antibody (C, D). Row A is at 2.5wPTI. Rows B-D are at 3wPTI, and row E at 4wPTI. **F**, BB of injected controls (no Cre), injected heterozygotes (with Cre), uninjected control and ^tam^*Cep164*^-/-^ retina assayed for presence of CEP164 immunoreactivity. Essentially all control BB displayed CEP164; in ^tam^*Cep164*^-/-^ retina, about 40% of BB did not. ^tam^*CEP164*^-/-^ is P < 0.05 compared to both controls. Numbers in columns refer to animal numbers (n = 3 each).

To explore consequences of CEP164 depletion in cones, we deleted *Cep164* in *Cep164*^F/F^;Prom1-CreER^T2^ one-month old mice by tamoxifen induction. PROM1 expression in adult mice occurs in rod/cone photoreceptors along with cells of the brain, pancreas, intestine/colon, kidney, lung and reproductive system (male and female) [25]. *Cep164*^F/+^;Prom1-CreER^T2^ mice (**Fig 9**, left column), *Cep164*^+/+^;Prom1-CreER^T2^ mice (**Fig 9**, middle column) and *Cep164*^F/F^;Prom1-CreER^T2^ mice (^tam^*Cep164*^-/-^) (**Fig 9**, right column) were injected with tamoxifen at ages ranging from P28 to P30. Intraperitoneal injection of tamoxifen for five consecutive days induced the nuclear translocation of *Cre*, and the degeneration rate was assessed in retina cryosections of eyes harvested 2–4 weeks post-tamoxifen induction (2-4wPTI) after the first injection.

**Fig 10:**
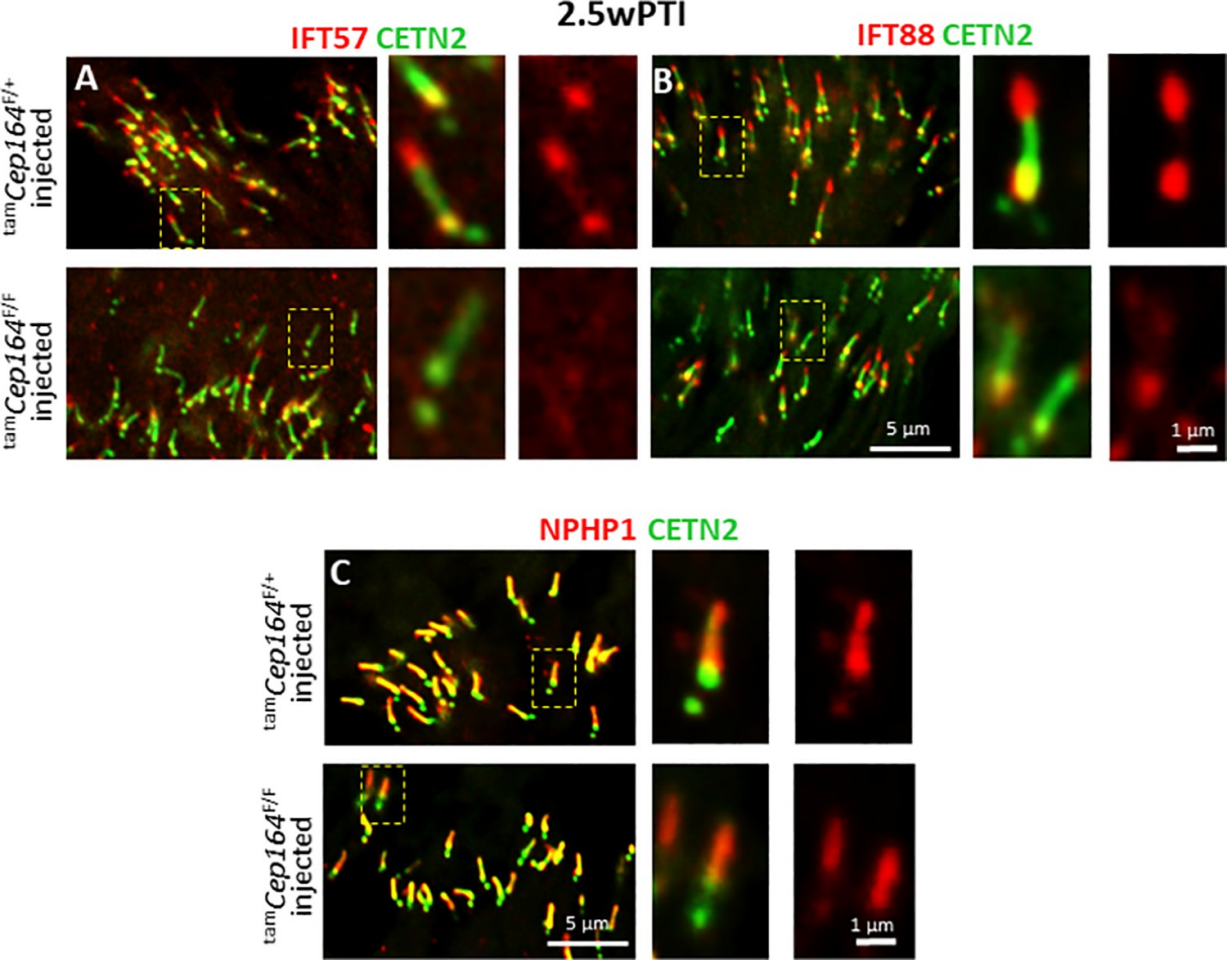
Immunohistochemistry with anti-IFT57, anti-IFT88 and anti-NPHP1 in ^tam^*Cep164*^-/-^ mice. **A-C,** immunohistochemistry of 2.5wPTI ^tam^*Cep164*^+/-^ (upper panels) and ^tam^*Cep164*^-/-^ (lower panels) photoreceptors probed with anti-IFT57 (red) (A), anti-IFT88 (B) and anti-NPHP1 (C) antibodies. Fluorescent signals of IFT57 and IFT88 immunolabeling are reduced in the knockout, NPHP1 is stably bound to the CC. Insets (right) show enlargements of representative BB/CC structure identified by Egfp-CETN2 (green) or individual antibodies (red).

Labeled by anti-PDE6 antibody (MOE), ^tam^*Cep164*^-/-^ rod OSs degenerated slowly and were approximately half-length at 3wPI (**Fig 9A**, right panel) relative to control OSs (**Fig 9A,** left and middle panels). ^tam^*Cep164*^-/-^ rod OSs probed with anti-ARL13B (**Fig 9B,** right panel) revealed that ARL13B is unable to reach the OS in the absence of CEP164, confirming the interaction of these two proteins [22]. ^tam^*Cep164*^-/-^ cone OSs identified by anti-ML-opsin were shorter at 3wPTI (**Fig 9C,** right panel) compared to controls (**Fig 9C,** left and middle panels) and ML-opsin mislocalized in part to the cone IS. At 4wPTI, mutant cone OSs were largely degenerated (**Fig 9D**, right panel). About 60% of ^tam^*Cep164*^-/-^ rods analyzed by confocal microscopy at 2.5wPTI had BBs associated with CEP164, while the rest were CEP164-free (n = 3, **Fig 9E** and **9F**) indicating a 40% induced knockout efficiency. Anterograde IFT57 and IFT88 showed normal distributions at control BB and CC tips (**Fig 10A** and **10B,** upper panels) (**Fig 6A** and **6B**). As expected, IFT57 and IFT88 were absent at the ^tam^*Cep164*^-/-^ CC tip and reduced at BB (**Fig 10A** and **10B,** lower panels), similar to the CEP164 rod knockout at P16. NPHP1 localization at CC was unaffected (**Fig 10C**).

**Fig 11:**
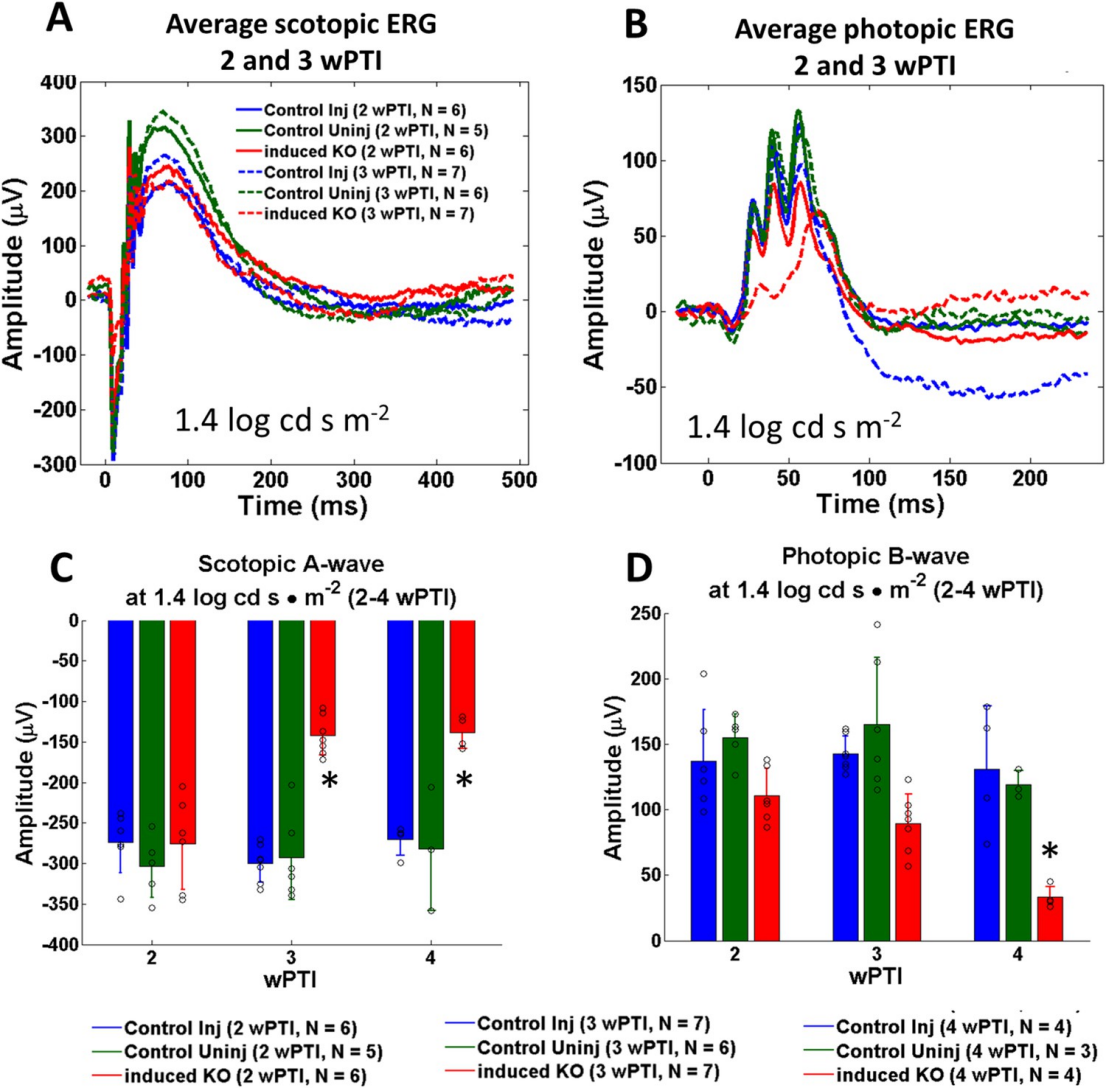
Electroretinography of control and ^tam^*Cep164*^-/-^ mice. **A, B**, average scotopic (A) and photopic ERG (B) at 1.4 log cd s m^-2^. 2wPTI is shown in solid traces, and 3wPTI in hatched traces. Control-injected traces (blue), control-uninjected traces (green), ^tam^*Cep164*^-/-^ (red). Traces are averaged with N = 5–7. **C, D**, statistical evaluation of scotopic a-waves (C) and photopic b-wave amplitudes (D) at 2wPTI, 3wPTI and 4wPTI at 1.4 log cd s/m^2^. Colors as in A and B, number of animals (N) used is shown at the bottom. * p<0.05, ANOVA.

Average ^tam^*Cep164*^*-/-*^ scotopic a-wave amplitudes, not significantly reduced at 2wPTI, were diminished by 3wPTI (n = 5–7, **Fig 11A**) indicating loss of rod function. Statistical evaluation of scotopic a-waves at 2-4wPTI at 1.4 log cd s/m^2^ confirmed diminished a-wave amplitudes (50% and 45%, respectively) (n = 3–7, **Fig 11C**). A-wave amplitudes did not significantly change at a higher light intensity of 2.4 log cd s/m^2^ for 2wPTI (n = 3–7, **S7A** and **S7B Fig**). Photopic ^tam^*Cep164*^*-/-*^ b-waves persistently diminished from 3wPTI down to 40% of control at 4wPTI (**Fig 11B** and **11D**). A- and b-wave amplitudes were not reduced further at 6wPTI and 9wPTI (**S7C and S7D Fig**). Pan-retina ERG as a function of light intensity (-4.5 to 2.4 log cd s/m^2^) did not reveal significant differences of scotopic/photopic a- and b-wave amplitudes at 2wPTI (**S8A–S8C Fig**). At 3wPTI, scotopic ERG showed diminished a- and b-wave amplitudes beginning at 0.6 log cd s/m^2^. Photopic b-wave amplitudes of control and ^tam^*Cep164*^*-/-*^ mice were statistically identical at 2wPTI, but decreased by 3wPTI (**S8C Fig**).

## Discussion

Many cell culture studies address the role of CEP164 during BB docking and early ciliogenesis [2,12,31,42] where disruption of CEP164 blocked primary cilium formation [12,51]. Similar results were obtained in zebrafish [12,13,16] and mouse [1,17,52]. Here, we describe effects of CEP164 deletion at three different stages of retina development by conditional recombination using Cre drivers which express Cre beginning at E9 (Six3Cre) [23], in the second postnatal week (iCre75) [24] or in the adult mouse (tamoxifen injection). Six3Cre-driven deletion of CEP164 has no effect on pre-ciliogenesis photoreceptor differentiation or ONL lamination (**Fig 1N**), but prevents OS formation (**Fig 2B** and **2E**), essentially confirming the well-documented role of CEP164 in cultured cells. Loss of CEP164 early leads to rapid rod and cone degeneration centrally with an LCA-like phenotype (**Fig 2G–2J**).

Because CEP164 disruption results in variable patient phenotypes, we reasoned that CEP164 may have additional functions to be revealed during post-ciliogenesis deletion of CEP164. Two drivers express Cre after ciliogenesis (iCre75 and tamoxifen inducible Prom1-CreER^T2^) when CEP164 is bound to distal appendages and the BB is docked to the apical membrane. Postnatal depletion of CEP164 in rods with iCre75 allows the BB to dock to the cell cortex. Control photoreceptor CC extend axonemes around P6, and OSs are mature at P21. P16 ^rod^*Cep164*^*-/-*^ plastic sections do not reveal major morphological changes compared to controls, but the ONL is reduced at P21 (**S4 Fig**), confirmed by statistical analysis comparing control and ^rod^*Cep164*^*-/-*^ ONL thickness (**Fig 3E**). At P30, only one nuclear row is present in the knockout (**Fig 3B and 3D**). PDE6 mislocalization in the IS was not obvious in the P21 ^rod^*Cep164*^*-/-*^ photoreceptors when OSs are disintegrating (**Fig 3B**), suggesting continuous delivery of PDE6 by its chaperone PDE6D to OSs as OS discs continue to be formed. By contrast, ARL13B is absent in ^rod^*Cep164*^*-/-*^ OSs consistent with the observation that ARL13B interacts with CEP164 (**Fig 3D**) [22]. The results suggest that the BB-CC-axoneme structure at the OS base is relatively stable even as the OS degenerates, in contrast to CEP164’s ciliogenesis role where CEP164 is required for recruitment and physical attachment of small preciliary vesicles to the mother centriole and membrane docking. While P16 control and ^rod^*Cep164*^*-/-*^ retina morphologies are comparable (**S4A Fig**), the scotopic a-wave already is reduced at P15 indicating rod degeneration has already begun (**Fig 5B**), although not statistically significant at this age. In P15 and P21 ^rod^*Cep164*^*-/-*^photoreceptors, most BB have lost CEP164 (**Fig 4B** and **4B**) but P16 OSs still possess BB-CC structures (**Fig 3B**).

A major finding is that IFT88, IFT57 and IFT140 are depleted gradually after deletion of CEP164 at P16-P21 (**Figs 6** and **S5**), implicating IFT malfunction as the cause of retina degeneration in ^rod^*Cep164*^*-/-*^ mice. Similar results were seen in tamoxifen-induction experiments at 2.5WPTI (**Fig 10A** and **10B**). Changes in localization and expression levels of PCNT and ninein **(S6 Fig**) suggest that other PCM and subdistal DAPs may be affected by CEP164 deletion. IFT is integral for assembly, maintenance, and function of motile and primary cilia [53,54], relying on molecular motors and IFT particles to move cargo along microtubules in anterograde (heterotrimeric kinesin-2) or retrograde (dynein-2) direction. IFT88 (Tg737) and IFT57 are required for ciliary and *Chlamydomonas* flagella assembly [55]. IFT57 stabilizes the IFT-B complex in *Chlamydomonas* flagella [56] and interacts with IFT20 and the kinesin-2 subunit, KIF3B [57]. Germline deletions of IFT88 are embryonically lethal [58,59]. In mouse photoreceptors, a hypomorphic mutation in the IFT88 gene exhibited abnormal OS development and retinal degeneration demonstrating that IFT88 is important for OS maintenance [32]. In conditional retina IFT88 knockouts (^ret^*Ift88*^-/-^), BBs failed to extend CC [60]. Loss of IFT88 (Tg737) and IFT57 (Hippi) during CEP164 deletion confirms CEP164’s involvement in recruiting these proteins to the photoreceptor BB, first observed in primary cilia [31]. Another DAP protein involved in recruiting IFT-B proteins (IFT88 and IFT52) is C2CD3 (OFD14) which localizes to the distal ends of both mother and daughter centrioles. C2CD3 was shown to recruit IFT88 and IFT52 to the mother centriole [4]. As C2CD3 is required for the recruitment of DAP proteins including CEP164, the effect of recruiting IFT-B proteins may be indirect. Loss of C2CD3 results in shortened centrioles without appendages and failure of CP110 removal from the ciliary mother centriole, a critical step in initiating ciliogenesis. This provides a potential mechanism for the later onset of retinal degeneration in patients with hypomorphic CEP164 mutations.

Instability of the OS distal tip with continued disc morphogenesis proximally was also seen in the CF-568 dye assay that allows monitoring of disc morphogenesis, enclosure and displacement in mouse rods (**Fig 8**). Intravitreally-injected CF-568-hydrazide [48] is sealed into the disc lumen during the disc enclosure process. The number of labeled discs, limited by available dye, is fewer than 80, the number of discs generated per day. Although disc morphogenesis continued as late as P18, the rate of disc renewal was impaired (**Fig 8E)**. We propose that instability at the ^rod^*Cep164*^-/-^ OS distal tip may be caused by axonemal disintegration as IFT-B proteins dissociate from BB thereby impairing IFT (**Figs 6** and **8C**).

IFT cargo in rods is controversial. Current models suggest that rhodopsin and other integral membrane proteins traffic by anterograde IFT, mediated by kinesin-2, along the CC for deposition into nascent discs [61–64]. In our hands iCre75-based conditional knockout of KIF3A, the kinesin-2 obligatory subunit, did not prevent trafficking of rhodopsin and other proteins to the OS [65]. Conditional knockout of KIF3A with Six3Cre prevented CC and axoneme formation, although BB docked to the cell membrane correctly [60]. Conditional knockout of KIF3A and IFT88 in the adult mouse by tamoxifen injection demonstrated that photoreceptor axonemes disintegrate slowly post-induction, starting distally, but rhodopsin and cone pigments traffic normally for more than 2 weeks, a time interval during which the OS is completely renewed. Thus, visual pigments likely transport to the photoreceptor OS despite KIF3A, IFT88 removals and IFT impairment. Alternatively, kinesin-2-mediated anterograde IFT appears to be responsible for photoreceptor transition zone and axoneme formation [60].

CEP164 forms a complex with PDEδ (a prenylated protein chaperone), INPP5E (a farnesylated inositol polyphosphate 5 phosphatase) and ARL13B (GEF of ARL3) to traffic prenylated INPP5E to primary cilia in cell culture [22]. However, INPP5E of WT mouse photoreceptors localizes to the IS, is excluded from the OS, and is apparently independent of PDEδ [66]. Absence of INPP5E in the OS would seem to nullify experiments to show that ARL13B, PDEδ or CEP164 act as chaperones for INPP5E. Connecting cilia and short rudimentary OSs form in ^ret^*Inpp5e*^-/-^ photoreceptors, but without axonemal structure and discs [66]. ARL13B localizes to WT OSs, but in ^ret^*Arl13b*^-/-^ [67] and ^ret^*Cep164*^-/-^ photoreceptors (**Fig 2E**), CC and OS fail to form and ARL13B accumulates in the IS. In ^rod^*Cep164*^-/-^ photoreceptors, OSs form but ARL13B is barely detectable at P16 and absent at P21 (**Fig 3D**), while in ^tam^*Cep164*^-/-^ photoreceptors, ARL13B is depleted in OS (**Fig 9B**, right panel). These observations are consistent with [22] who show that ARL13B and CEP164 interact to enable ARL13B to reach the cilium, but not with [17] where ARL13B accumulated in CEP164-KO multiciliated cells. Thus, the functional network consisting of ARL13B, INPP5E, PDEδ and CEP164 is an arrangement of primary cilia for INPP5E and ARL13B to traffic to cilia, but is conserved only partially in photoreceptors.

### Summary

Deletion of CEP164 in photoreceptors before ciliogenesis prevented BB docking as shown in primary cilia, leading to rapid rod/cone degeneration resembling LCA. Deletion of CEP164 after ciliogenesis and BB docking reveals that BB/CC/OS structure lacking CEP164 is initially stable. Post-ciliogenesis deletion of CEP164 causes loss of IFT proteins and impairment of IFT, an essential transport mechanism for OS assembly and maintenance. Impairment of IFT is demonstrated by a fluorescent dye assay that labels newly generated discs. In ^rod^*Cep164*^-/-^ rods, discs that travel to the mid-distal OS degenerate, while disc morphogenesis continues proximally at a slower rate, consistent with axoneme damage and IFT impairment. We favor a model in which the ^rod^*Cep164*^-/-^ axoneme is ill-maintained as IFT-B proteins dissociate from the BB, causing OS degeneration as IFT trains fail. Loss of IFT88 and IFT57 during CEP164 deletion suggests that CEP164 recruits anterograde IFT proteins to the BB as proposed by [31], and identifies CEP164 and DAP structure as integral for initiating IFT.

## Materials and methods

### Ethics statement

All mice were handled in accordance with NIH guidelines, and all protocols were approved by the Institutional Animal Care and Use Committee (IACUC) of the University of Utah (Protocol 18–11005).

### Animals

Prom1^tm1(cre/ERT2)Gilb^ (Stock No: 017743) and Egfp-*Cetn2* mice Stock No. 008234—CB6-Tg(CAG-EGFP/CETN2)3-4Jgg/J) were obtained from The Jackson Laboratory. iCre75 mice were generated in Utah [24]. *Cep164*^F/F^ mice were described previously [17].

### Generation of retina- and rod-knockout mice

*Cep164*^F/F^ mice were crossed with Six3Cre [23] or iCre75 transgenic mice [24] kept on Egfp-Cetn2 background to generate *Cep164* ^F/+^;Six3Cre;Egfp-*Cetn2* (^ret^*Cep164*^+/-^) or *Cep164* ^F/+^;iCre75;Egfp-*Cetn2* (^rod^*Cep164*^+/-^) mice. Mice were then backcrossed to *Cep164*^F/F^ to generate experimental animals.

### Generation of knockouts by tamoxifen induction

*Cep164*^F/F^ mice were bred to Prom1-CreER^T2^ mice [25] to generate *Cep164*^F/+^;Prom1-CreER^T2^ mice. Expression of CreER^T2^ is driven by the prominin 1 (Prom1) promoter/enhancer. *Cep164*^F/+^;Prom1-CreER^T2^ mice were bred to *Cep164*^F/F^ mice to generate mice for tamoxifen injection. Tamoxifen was administered via intraperitoneal injection in adult mice (P28-P30 at time of first injection). Tamoxifen was dissolved in corn oil to a stock solution of 20mg/ml. Mice were dosed with 150 mg/kg body weight for 5 consecutive days according to their weight on the first day of injection (i.e., 7.5 μL of 20mg/ml tamoxifen solution per gram weight). ERG and eye collection for confocal immunolocalization were performed 2–9 weeks after the first injection.

### Genotyping

Genomic DNA was extracted from fresh tissue by dissolving tail clips or retina DNA from P8-12 day old mice in 150 μL tail lysis buffer at 50–60°C for 1–2 hour. Digests were centrifuged at 15000 rpm for 5 minutes. Supernatant was added to an equal volume of isopropanol and centrifuged at 15000 rpm for 5 minutes. The DNA pellet was rehydrated in 75 μL H_2_O. Genotyping was achieved by polymerase chain reaction with EconoTaq DNA polymerase (Lucigen). Primers for genotyping of CEP164 mice: 5’-CCATCTGTCCAGTACCATTAAAAA and 5’-CCCAGAATACAACATGGGAGA (WT allele, 215 bp; floxed allele, 415 bp). The CEP164 exon 4 excision assay used CEP164 floxed allele F1 primer, 5’-CCATCTGTCCAGTACCATTAAAAA and R2 primer 5’-GACAAGTTCCATCTACCACAATC (WT allele 1525 bp; Floxed allele 1696 bp; Exon 4 excision allele 719 bp).

### Dye injection

CF-568-hydrazide is a water soluble, membrane impermeable, aldehyde-fixable red-fluorescent dye [48]. Heterozygous control (^rod^*Cep164*^F/+^;Egfp-Cetn2) and knockout (^rod^*Cep164*^F/F^;Egfp-Cetn2) eyes were injected intravitreally with 1.5 μL of 0.5% CF-568-hydrazide [48] in 1x PBS on P14 and P18, and retinas were harvested on P19, at which time about half OS-length has been replaced. Treated retinas were dissociated mechanically and images acquired using a Zeiss LSM 800 microscope.

### Immunohistochemistry

Eyes were embedded without fixation, or fixed 10 minutes or 1 hour in 4% paraformaldehyde (PFA) in 0.1M phosphate buffer (pH 7.4) and embedded in OCT (Fisher). Sections were allowed to adhere to slides for 30 minutes at 37°C. Eyes embedded without initial fixation had the OCT compound dissolved by adding a small volume of 1X PBS, holding stationary for 5 minutes at room temperature, and then adding 1:1 methanol-acetone for 10 minutes at 4°C. Slides were rehydrated by washing either 10 minutes (X3) or 5 minutes (X4) in 1X PBS. All sections used were from the central retina near the optic nerve, except when labeled as peripheral. Sections were blocked in 5% normal goat serum (NGS); 0.1% Triton in 1X PBS (for 1 hour 4% PFA fixation) or 5% NGS in 1X PBS (for 10 minutes 4% PFA or 10 minutes methanol-acetone fixation) for 1 hour. Antibodies, fixation, dilutions and sources: PDE6 (1 hour 4% PFA, 1:1000; MOE Cytosignal) [64]; OPN1MW/OPN1LW (1 hour 4% PFA, 1:500, Millipore Sigma AB5405) [65]; CEP164 (10 m minutes in 4% PFA, 1:350, Sigma-Aldrich); ARL13B (1 hour 4% PFA,1:350, ProteinTech); IFT57 (10 minutes 4% PFA, 1:200, Pazour lab); IFT88 (10 minutes 4% PFA, 1:200, Pazour lab); IFT120 (10 minutes 4% PFA, 1:150, Pazour lab); SPATA7 (10 minutes 4% PFA, 1:100, Chen lab, purified as described [35]); CEP290 (10 minutes 4% PFA, 1:300, Swaroop laboratory); PCNT (10 minutes 4% PFA, 1:250, Covance); Ninein (10 minutes 4% PFA, 1:100, Abclonal and Sigma-Aldrich); NPHP1 (10 minutes 4% PFA, 1:100, [68]); FBF1 (10 minutes methanol-acetone fix, 1:200, Proteintech). Primary antibodies were diluted in blocking buffer and applied to sections; sections were then incubated overnight at 4°C. Slides were washed for 10 minutes (X3) or 5 minutes (X4) in 1X PBS. Secondary antibodies were diluted in blocking buffer (goat anti-rabbit Alexa Fluor 555, 1:1000 (Invitrogen 32732); goat anti-mouse Alexa Fluor 555, 1:1000 (Invitrogen 32737); DAPI, 1:3000), applied to the sections and incubated with rotation, in the dark, for 1 hour at room temperature. Slides were washed for 10 minutes (X3) or 5 minutes (X4) in 1X PBS. Slides were dipped briefly in deionized H_2_O, and coverslipped using Fluoromount-G Mounting Medium (Southern Biotech). Images were acquired using a Zeiss LSM 800 confocal microscope with 63X objective and post-processed with Airyscan. All genotypes of a given age and antibody were imaged at a single z-plane using identical settings for laser intensity and master gain. Digital gain was 1 for all images. Pinhole size was set for 1AU on the red channel (39 μm for the 40X objective). Post-processing of non-saturated images consisted of equal adjustments to brightness/contrast of control and knockout images using Adobe Photoshop but without affecting the conclusions. Red channel separation was obtained by isolating the R-channel in the “blender options” of Adobe Photoshop.

### Intensity measurements

We used the average pixel intensity of a drawn region of interest (ROI) function in Zen Blue. For IFT markers, a circle ROI was drawn around the distal tip of the CC, as determined by the Cetn2-EGFP signal. Over 30 cells were counted across 3 different confocal images and averaged per mouse. For CC markers, a rectangular ROI was drawn around the CC and average intensity measured. We performed a two-tailed Student’s t-Test to compare controls to knockouts. Microsoft Excel function "T.TEST assuming equal variances, two-tailed" was used to calculate the p-value, with statistical significance determined using an alpha value of p < 0.05.

### Electroretinography

Scotopic and photopic ERG measurements were performed using P16 and P25 for Six3Cre experiments and P16, P21, or P30 mice for the iCre75 experiments and at 2, 3, 4, 6 or 9 weeks post-tamoxifen induction for the Prom1-CreER^T2^ experiments. Prior to ERG the mice were dark-adapted overnight and anesthetized with intraperitoneal injection of 1% ketamine/0.1% xylazine at 10 μl/g body weight. The mice were kept warm during ERG by using a temperature-controlled stage. Scotopic and photopic responses were recorded as described [66] using a UTAS BigShot Ganzfeld system (LKC Technologies, Gaithersburg, MD). Scotopic single-flash responses were recorded at stimulus intensities of -4.5 log cd s·m^-2^ [log candela seconds per square meter] to 2.4 log cd s·m^-2^). Mice were light-adapted under a background light of 1.48 log cd s·m^-2^ for 5 minutes prior to measuring photopic responses. Photopic single-flash responses of control and knockout were recorded at stimulus intensities of -0.1 log cd s·m^-2^ to 1.9 log cd s·m^-2^.

### Statistical analysis

We performed an unbalanced two-factor ANOVA to compare mutant and control mice for their quantified a- and b-wave ERG response across multiple ages or weeks post-tamoxifen induction. Post-hoc multiple comparison was performed using Tukey’s honestly significant difference criterion. Statistical significance was determined using an alpha value of p < 0.05. ERG statistics were computed using MATLAB’s statistical toolbox "anovan" and "multcompare" functions.

We performed a two-tailed Student’s *t*-Test assuming equal variances to compare ONL thickness for ^ret^*Cep164*^+/-^, ^ret^*Cep164*^-/-^, ^rod^*Cep164*^+/-^ and ^rod^*Cep164*^-/-^. Retina measurements used for these calculations were determined based on an average of three measurements per retina. Microsoft Excel function "T.TEST assuming equal variances, two-tailed" was used to calculate the p-value, with statistical significance determined using an alpha value of p < 0.05.

## Supporting information

S1 Fig CEP164 localizes to BB docked at the inner segment membrane. **A**, immunolocalization of CEP164 (red) in P21 *Cep164*^+/+^;Egfp-Cetn2 (green) photoreceptors. CEP164 is an inner segment protein associating with basal bodies (BBs). **B**, BB enlargement with connecting cilium (CC) and daughter centriole (DC). Expression of transgenic Egfp-CETN2 identifies the CC, BB (= mother centriole, MC) and DC. **C**, BB docking pathway, ciliogenesis and IFT. **a**, centrosome consisting of MC and DC; MC and DC distal ends are capped by CP110/CEP97 (green). **b**, MC acquires distal appendage proteins (DAPs, light blue) and CEP164 (red). **c**, TTBK2 (tau tubulin kinase 2) phosphorylates DAPs which removes the terminal cap and allows docking. MC matures to BB. **d**, BB cap removal permits extension of A- and B-tubules to form an axoneme and transition zone (TZ). **e**, BB/TZ acquire IFT proteins and kinesin-2 to assist IFT. **f**, IFT delivers subunits to extend the axoneme and initiate disc morphogenesis. **g**, deletion of CEP164 post-ciliogenesis leads to loss of IFT protein and cell death.
(TIF)

S2 Fig Generation of CEP164 conditional knockout mice. **A,** stages of rod photoreceptor ciliogenesis. E9 depicts a progenitor cell in which Six3Cre is expressed. At P0-P1, the centrosome consisting of mother (MC) and daughter (DC) centrioles acquires a ciliary vesicle (CV) distally. From P3-P4, the mother centriole docks to the cortex of the inner segment (IS). During P5-P6, an axoneme emanates from the MC and MC becomes a basal body (green). A- and B-tubules (black) extend at P7-P8, and the transition zone (TZ) becomes established. As plasma membrane evaginates and discs assemble, the rod outer segment (OS) is considered mature at three weeks of age (P21). Adapted from [21], with permission from Elsevier. **B,** gene structures of human centrosomal protein 164 kDa (*CEP164*; upper) and mouse *Cep164* (lower) consisting of a single WW (yellow) and three coiled-coiled (CC) domains. Positions of mutations associated with human nephronophthisis (NPHP) and retina degeneration are indicated. Asterisk above mouse CEP164 indicates the truncation point of our conditional knockout. **C,** schematic of *Cep164* exons 1–7 and conditional *Cep164* allele with loxP recombination sites flanking exon 4. The FRT site originates from flippase removing the gene trap. **D**, Cre-induced recombination (driven by Six3Cre, iCre75 or Prom1-ETCre) excises exon 4 and generates a null allele.
(TIF)

S3 Fig Genotyping. **A-E,** PCR amplification for the presence of WT and floxed Cep164 alleles (A), as well as Six3Cre (B), iCre75 (C), EGFP-CETN2 (D), and Prom1-ETCre (E) transgenes from tail DNA. **F**, verification of exon 4 excision using primers, F1 and R2, and P6 ^ret^*Cep164*^-/-^ retina DNA as template. **G**, rod knockout verification of exon 4 excision using primers, F1 and R2, and P16 ^rod^*Cep164*^-/-^ retina DNA as template. Lane 1, size markers; lane 2, +/F DNA; lane 3, F/F DNA; lane 4, +/F DNA with iCre75; lane 5, F/F DNA and iCre75; lane 6, water control. Note knockout (-/-) is complete in lane 5. **H**, genotyping of tamoxifen-induced CEP164 deletion at 2.5 weeks post-tamoxifen injection (2.5wPTI). Lane 1, +/F uninjected without Prom1EtCre. Lane 2, +/+ injected without Prom1ETCre. Lane 3, F/F injected with Prom1-ETCre (KO). Lane 4, water control. Note floxed exon 4 is still present and knockout is incomplete.
(TIF)

S4 Fig Cep164 rod knockout histology. **A, B,** retina morphology at P16 (A) and P21 (B). Note mutant OSs are still full-length at P16, but reduced by P21.
(TIF)

S5 Fig Red channel pixel intensity at P16. Pixel intensities of IFT88, IFT57 and IFT140 at ^rod^*Cep164*^-/-^ CC distal end were not significant, p>0.05. Three animals each were used.
(TIF)

S6 Fig Immunohistochemistry with anti-PCNT and anti-ninein. **A-C,** immunohistochemistry of P21 ^rod^*Cep164*^+/-^ (upper panels) and ^rod^*Cep164*^-/-^ cryosections (lower panels) probed with antibodies (red) directed against PCNT (A), ninein with Abclonal antibody (B) and Sigma-Aldrich antibody (C). Pericentrin locates to the pericentriolar material surrounding the BB (yellow); PCNT levels appear slightly reduced in ^rod^*Cep164*^-/-^. Ninein is a subdistal appendage protein associated with centrioles in tissue culture; in rods ninein is very weak at the BB but present in the proximal axoneme of control rods (B, upper panels) or rootlet/microtubule area (C, upper panels) (arrows in B and C enlargements). Panels to the right, enlargements of representative BB/CCs identified by EGFP-CETN2 (green) or individual antibodies (red).
(TIF)

S7 Fig Evaluation of scotopic a-wave and photopic b-wave amplitudes of tamoxifen-induced knockouts versus controls. **A-D**, statistical evaluation of scotopic a-wave (A, B) and photopic b-wave (C, D) amplitudes measured at light intensities of 1.4 log cd s/m^2^ (A, C) and 2.4 log cd s/m^2^ (B, D) at 2–9 wPTI. That OSs do not completely degenerate may be due to insufficient tamoxifen, or the PROM1 promoter unable to express higher levels of ETCre.
(TIF)

S8 Fig ERG measurements post-tamoxifen induction. **A-C,** amplitudes (μV) of scotopic a-wave (A), scotopic b-wave (B) and photopic b-wave (C) as a function of light intensity (log cd s m-^2^). Scotopic and photopic ERGs show statistically distinct responses at 3wPTI (^, p < .05, ANOVA at 2wPTI; *, p<0.05 ANOVA at 3wPTI).
(TIF)

S9 Fig Model of photoreceptor CEP164-mediated intraflagellar transport. **A,** during anterograde (directed toward the microtubule plus-end) IFT, kinesin-2 (KIF3A, KIF3B and KAP subunits) moves cargo along the proximal axoneme (microtubule doublet). Cargo likely consists of IFT-A and -B particles, retrograde dynein-2 motors, and axoneme building blocks and stabilizing factors. Mechanisms concerning axoneme maintenance is currently unclear. **B,** loss of IFT-A and -B particles, caused by CEP164 depletion, terminates IFT and photoreceptor OSs degenerate.
(TIF)
