# Supplementary figures and images for: Deletion of CEP164 in mouse photoreceptors post-ciliogenesis interrupts ciliary intraflagellar transport (IFT)
Source: PLoS Genet. 2022 Sep 8;18(9):e1010154. doi: 10.1371/journal.pgen.1010154 (PMC9488791; doi:10.1371/journal.pgen.1010154)

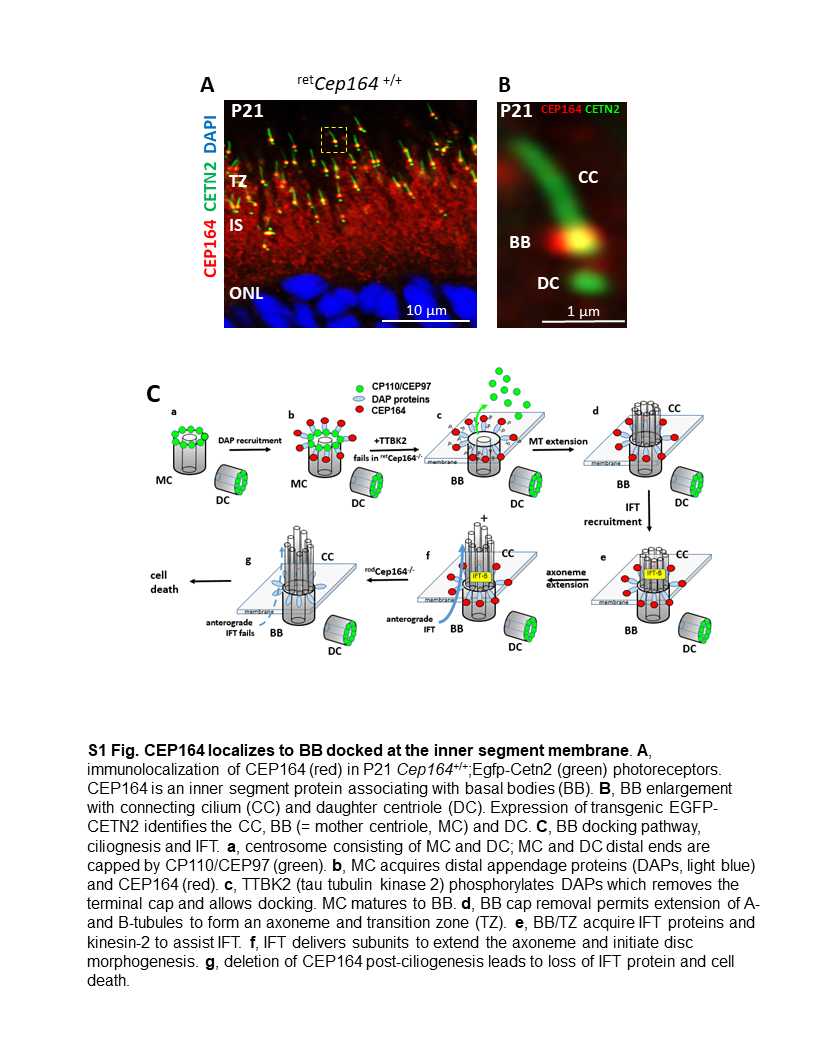

Supplement: S1 Fig — A, immunolocalization of CEP164 (red) in P21 Cep164+/+;Egfp-Cetn2 (green) photoreceptors. CEP164 is an inner segment protein associating with basal bodies (BBs). B, BB enlargement with connecting cilium (CC) and daughter centriole (DC). Expression of transgenic Egfp-CETN2 identifies the CC, BB (= mother centriole, MC) and DC. C, BB docking pathway, ciliogenesis and IFT. a, centrosome consisting of MC and DC; MC and DC distal ends are capped by CP110/CEP97 (green). b, MC acquires distal appendage proteins (DAPs, light blue) and CEP164 (red). c, TTBK2 (tau tubulin kinase 2) phosphorylates DAPs which removes the terminal cap and allows docking. MC matures to BB. d, BB cap removal permits extension of A- and B-tubules to form an axoneme and transition zone (TZ). e, BB/TZ acquire IFT proteins and kinesin-2 to assist IFT. f, IFT delivers subunits to extend the axoneme and initiate disc morphogenesis. g, deletion of CEP164 post-ciliogenesis leads to loss of IFT protein and cell death. (TIF) [file pgen.1010154.s001.tif]

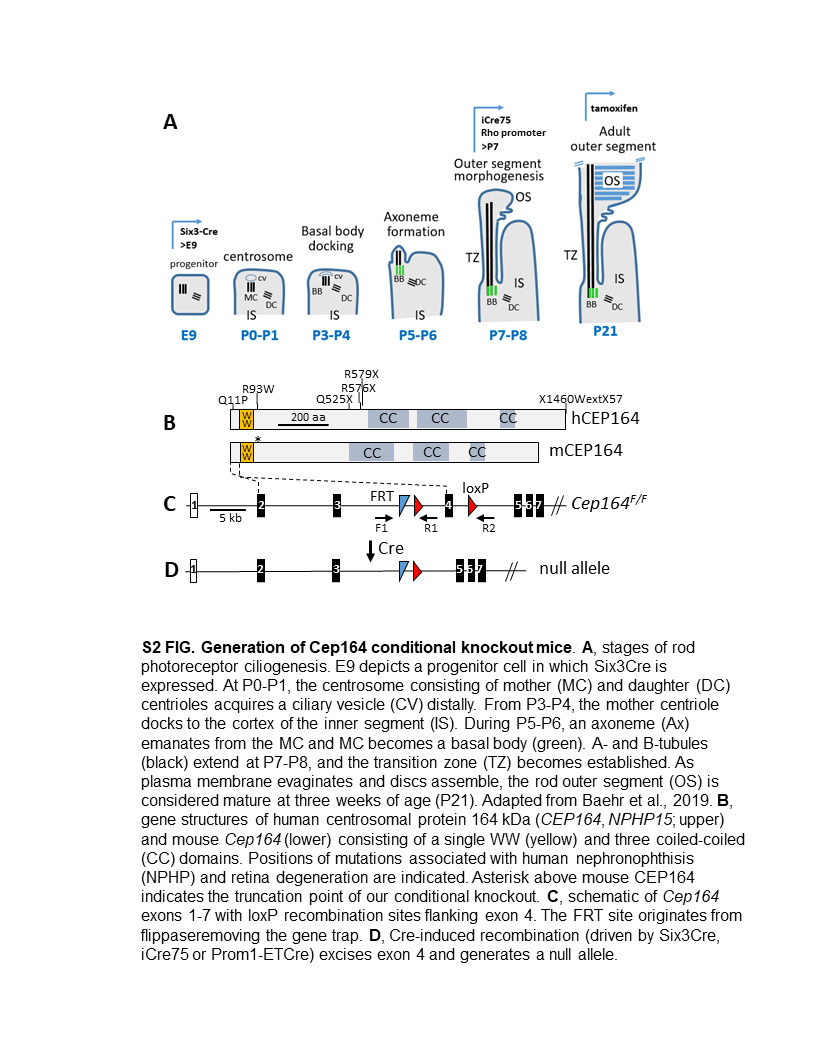

Supplement: S2 Fig — A, stages of rod photoreceptor ciliogenesis. E9 depicts a progenitor cell in which Six3Cre is expressed. At P0-P1, the centrosome consisting of mother (MC) and daughter (DC) centrioles acquires a ciliary vesicle (CV) distally. From P3-P4, the mother centriole docks to the cortex of the inner segment (IS). During P5-P6, an axoneme emanates from the MC and MC becomes a basal body (green). A- and B-tubules (black) extend at P7-P8, and the transition zone (TZ) becomes established. As plasma membrane evaginates and discs assemble, the rod outer segment (OS) is considered mature at three weeks of age (P21). Adapted from [21], with permission from Elsevier. B, gene structures of human centrosomal protein 164 kDa (CEP164; upper) and mouse Cep164 (lower) consisting of a single WW (yellow) and three coiled-coiled (CC) domains. Positions of mutations associated with human nephronophthisis (NPHP) and retina degeneration are indicated. Asterisk above mouse CEP164 indicates the truncation point of our conditional knockout. C, schematic of Cep164 exons 1–7 and conditional Cep164 allele with loxP recombination sites flanking exon 4. The FRT site originates from flippase removing the gene trap. D, Cre-induced recombination (driven by Six3Cre, iCre75 or Prom1-ETCre) excises exon 4 and generates a null allele. (TIF) [file pgen.1010154.s002.TIF]

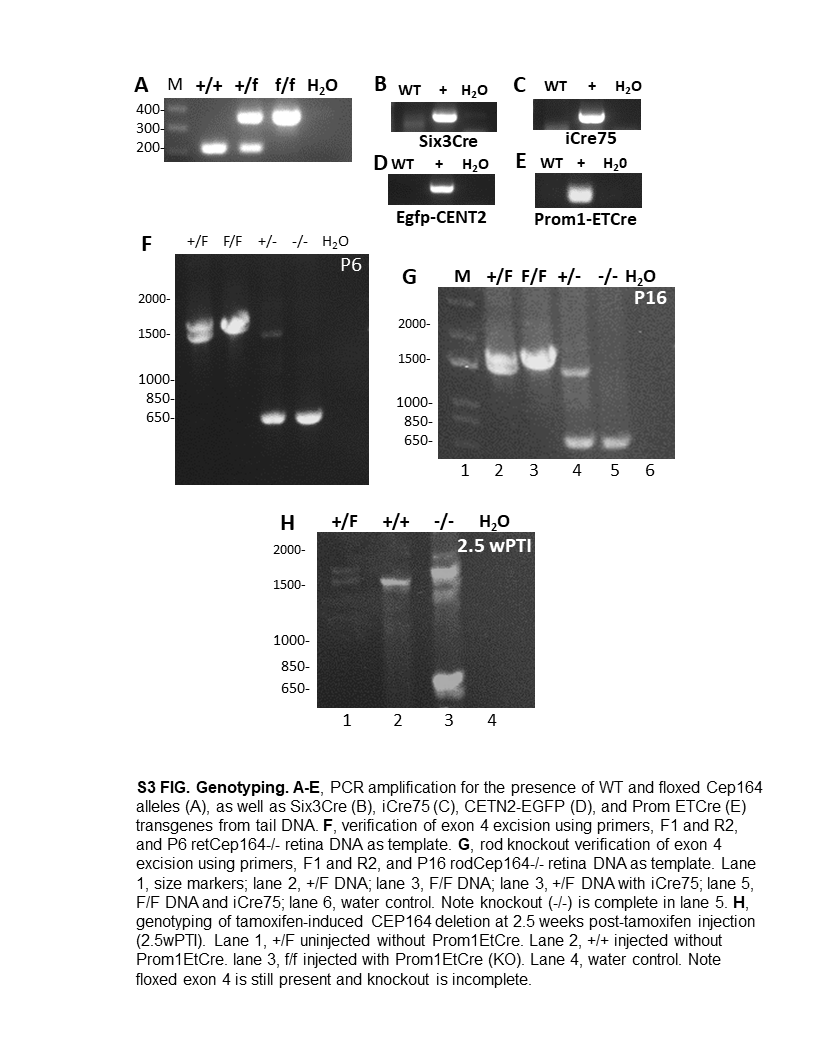

Supplement: S3 Fig — A-E, PCR amplification for the presence of WT and floxed Cep164 alleles (A), as well as Six3Cre (B), iCre75 (C), EGFP-CETN2 (D), and Prom1-ETCre (E) transgenes from tail DNA. F, verification of exon 4 excision using primers, F1 and R2, and P6 retCep164-/- retina DNA as template. G, rod knockout verification of exon 4 excision using primers, F1 and R2, and P16 rodCep164-/- retina DNA as template. Lane 1, size markers; lane 2, +/F DNA; lane 3, F/F DNA; lane 4, +/F DNA with iCre75; lane 5, F/F DNA and iCre75; lane 6, water control. Note knockout (-/-) is complete in lane 5. H, genotyping of tamoxifen-induced CEP164 deletion at 2.5 weeks post-tamoxifen injection (2.5wPTI). Lane 1, +/F uninjected without Prom1EtCre. Lane 2, +/+ injected without Prom1ETCre. Lane 3, F/F injected with Prom1-ETCre (KO). Lane 4, water control. Note floxed exon 4 is still present and knockout is incomplete. (TIF) [file pgen.1010154.s003.TIF]

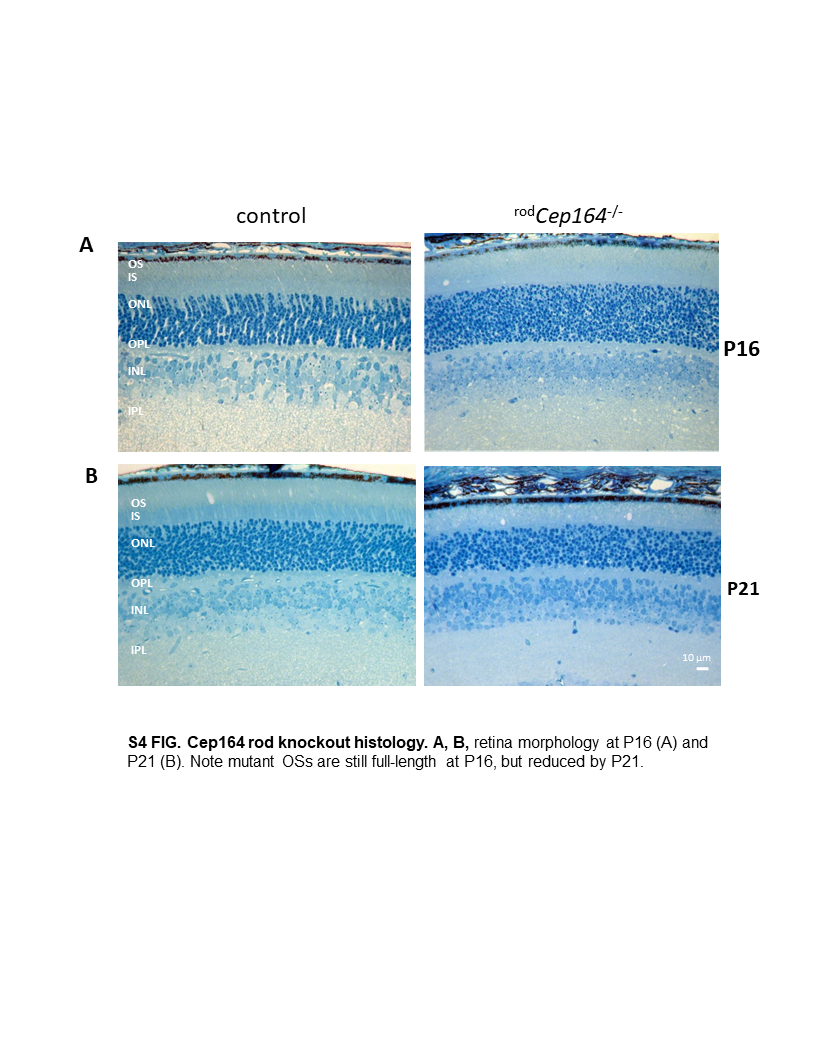

Supplement: S4 Fig — A, B, retina morphology at P16 (A) and P21 (B). Note mutant OSs are still full-length at P16, but reduced by P21. (TIF) [file pgen.1010154.s004.TIF]

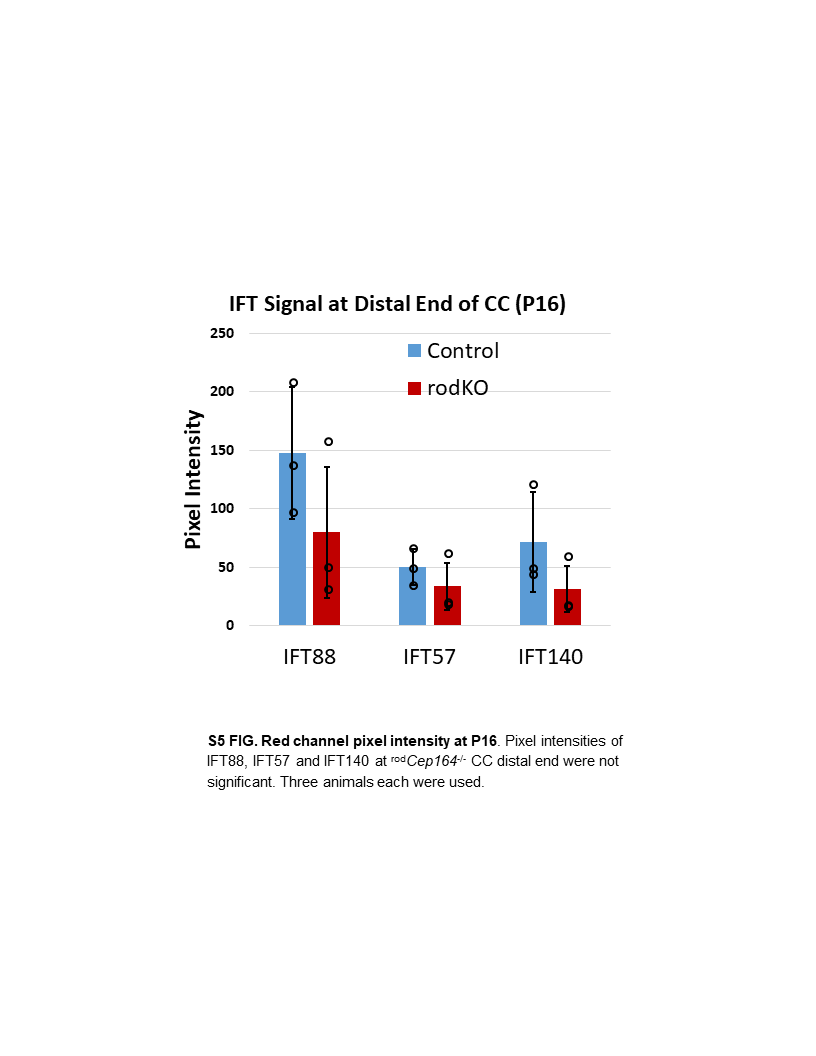

Supplement: S5 Fig — Pixel intensities of IFT88, IFT57 and IFT140 at rodCep164-/- CC distal end were not significant, p>0.05. Three animals each were used. (TIF) [file pgen.1010154.s005.TIF]

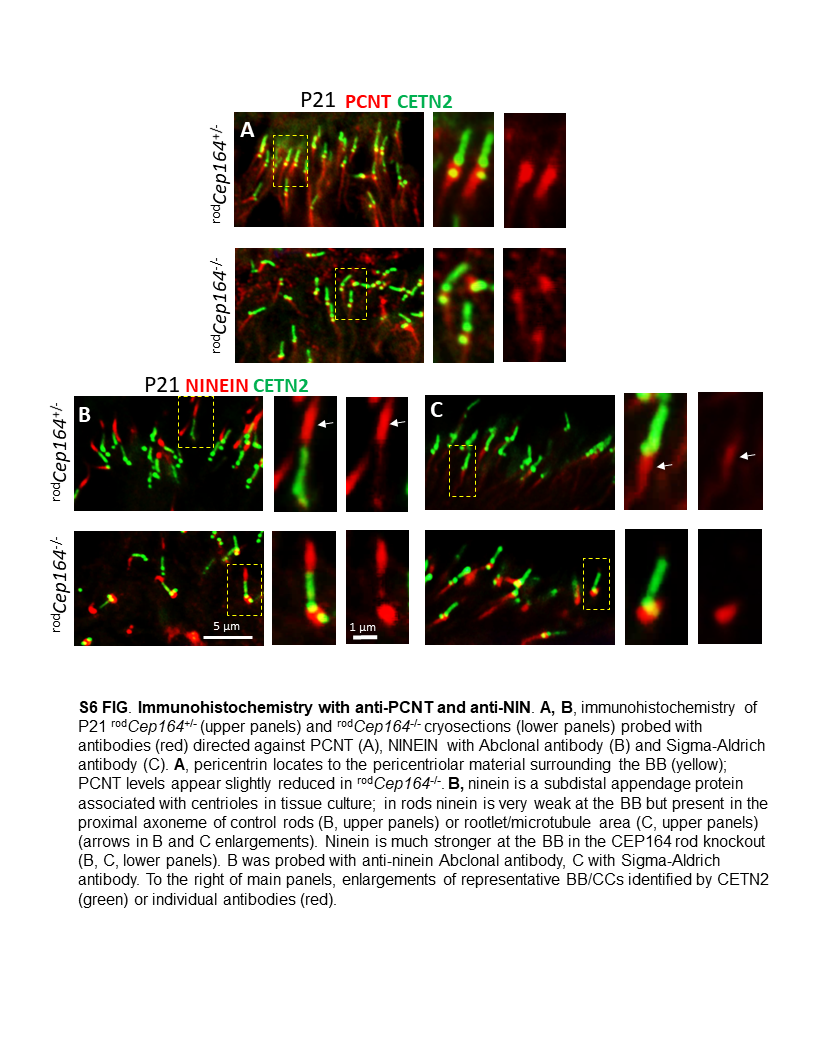

Supplement: S6 Fig — A-C, immunohistochemistry of P21 rodCep164+/- (upper panels) and rodCep164-/- cryosections (lower panels) probed with antibodies (red) directed against PCNT (A), ninein with Abclonal antibody (B) and Sigma-Aldrich antibody (C). Pericentrin locates to the pericentriolar material surrounding the BB (yellow); PCNT levels appear slightly reduced in rodCep164-/-. Ninein is a subdistal appendage protein associated with centrioles in tissue culture; in rods ninein is very weak at the BB but present in the proximal axoneme of control rods (B, upper panels) or rootlet/microtubule area (C, upper panels) (arrows in B and C enlargements). Panels to the right, enlargements of representative BB/CCs identified by EGFP-CETN2 (green) or individual antibodies (red). (TIF) [file pgen.1010154.s006.TIF]

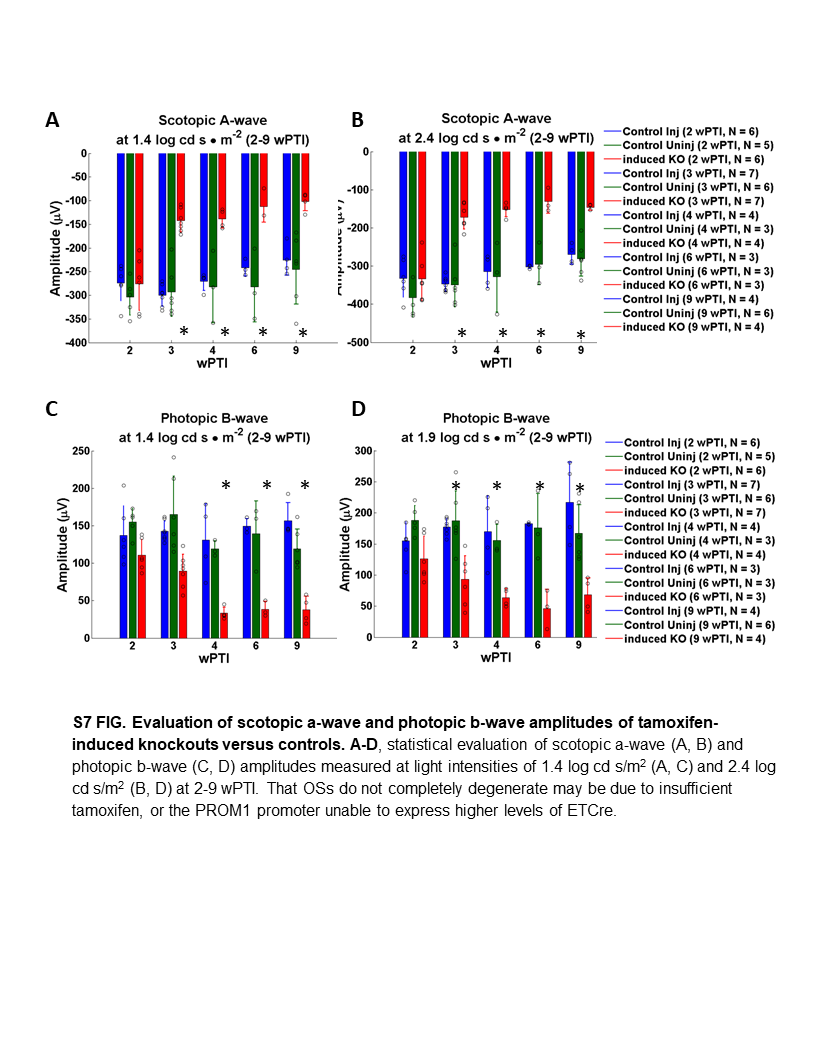

Supplement: S7 Fig — A-D, statistical evaluation of scotopic a-wave (A, B) and photopic b-wave (C, D) amplitudes measured at light intensities of 1.4 log cd s/m2 (A, C) and 2.4 log cd s/m2 (B, D) at 2–9 wPTI. That OSs do not completely degenerate may be due to insufficient tamoxifen, or the PROM1 promoter unable to express higher levels of ETCre. (TIF) [file pgen.1010154.s007.TIF]

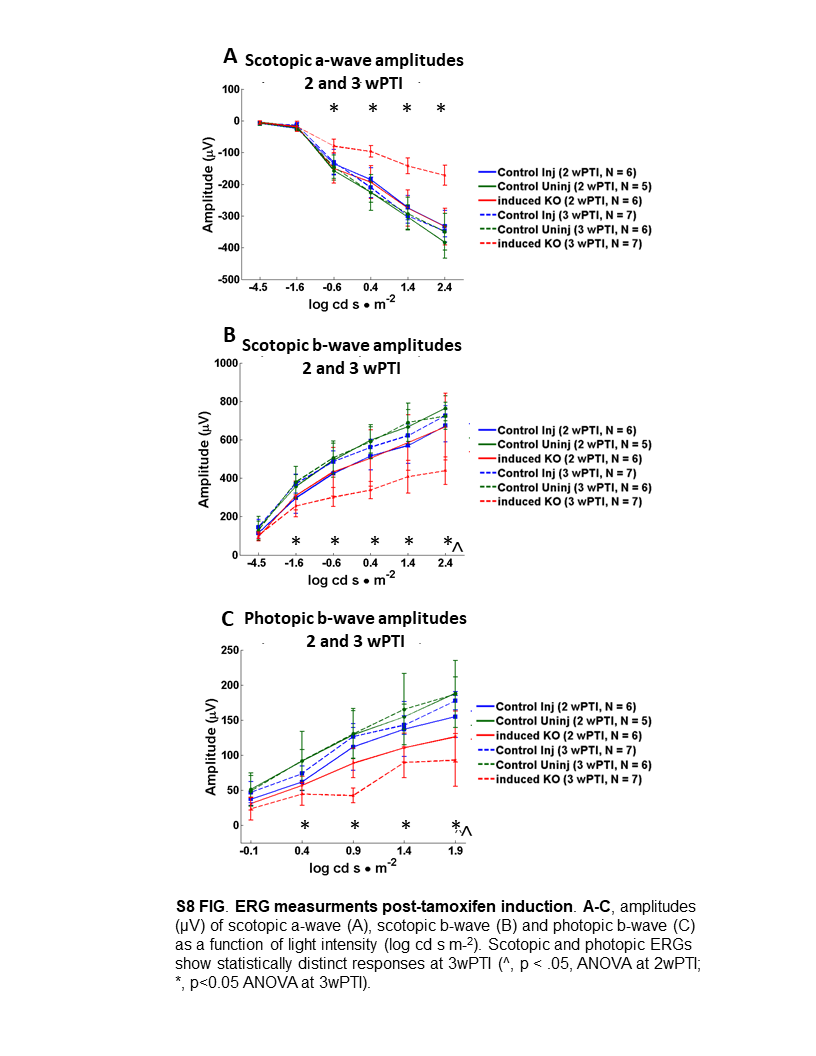

Supplement: S8 Fig — A-C, amplitudes (μV) of scotopic a-wave (A), scotopic b-wave (B) and photopic b-wave (C) as a function of light intensity (log cd s m-2). Scotopic and photopic ERGs show statistically distinct responses at 3wPTI (^, p < .05, ANOVA at 2wPTI; *, p<0.05 ANOVA at 3wPTI). (TIF) [file pgen.1010154.s008.TIF]

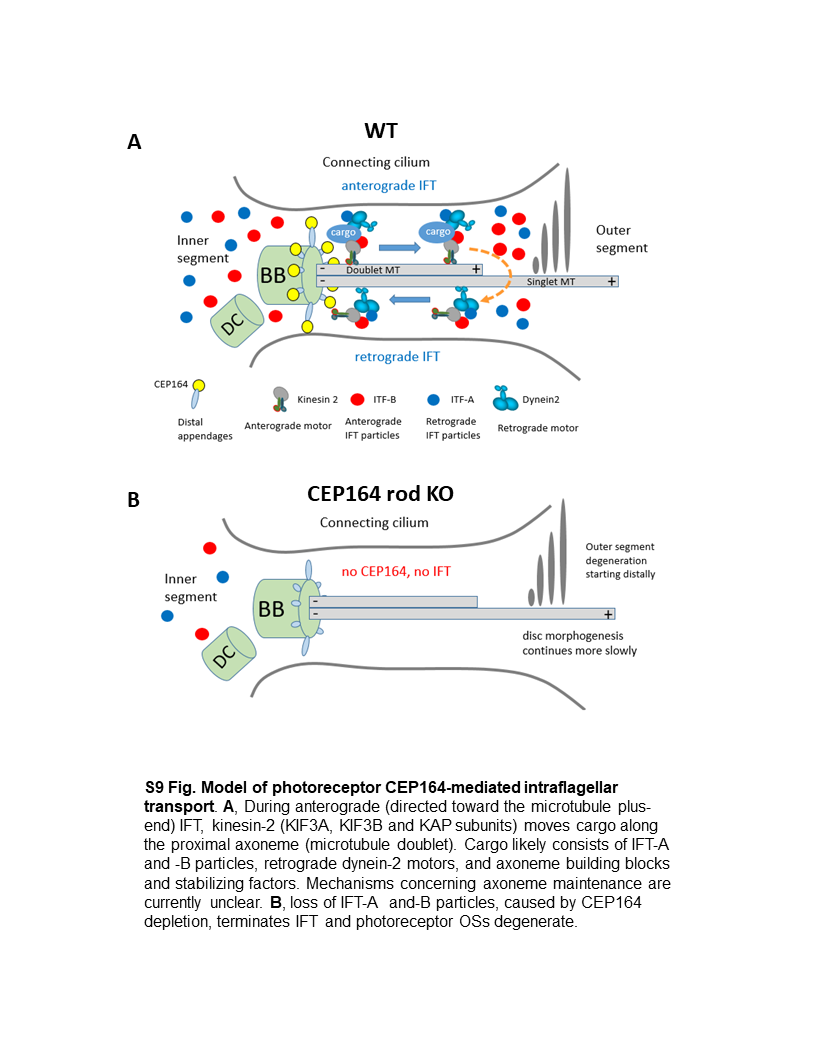

Supplement: S9 Fig — A, during anterograde (directed toward the microtubule plus-end) IFT, kinesin-2 (KIF3A, KIF3B and KAP subunits) moves cargo along the proximal axoneme (microtubule doublet). Cargo likely consists of IFT-A and -B particles, retrograde dynein-2 motors, and axoneme building blocks and stabilizing factors. Mechanisms concerning axoneme maintenance is currently unclear. B, loss of IFT-A and -B particles, caused by CEP164 depletion, terminates IFT and photoreceptor OSs degenerate. (TIF) [file pgen.1010154.s009.tif]
